# Multifunctional Dipoles Enabling Enhanced Ionic and Electronic Transport for High-Energy Batteries

**DOI:** 10.1007/s40820-025-01926-7

**Published:** 2026-01-05

**Authors:** Shihai Cao, Yuntong Sun, Yinghao Li, Ao Wang, Wenyao Zhang, Zhendong Hao, Jong-Min Lee

**Affiliations:** 1 School of Environmental Engineering, Nanjing Institute of Technology, Nanjing, 211167 People’s Republic of China; 2 School of Chemistry, Chemical Engineering and Biotechnology, Nanyang Technological University, 62 Nanyang Drive, Singapore, 637459 Singapore; 3 School of Materials Science and Engineering, Nanjing Institute of Technology, Nanjing, 211167 People’s Republic of China; 4 Key Laboratory for Soft Chemistry and Functional Materials, School of Chemistry and Chemical Engineering, Nanjing University of Science and Technology, Nanjing, 210094 People’s Republic of China; 5 Department of Energy Science and Engineering, Daegu Gyeongbuk Institute of Science and Technology (DGIST), Daegu, 42988 Republic of Korea

**Keywords:** High-energy batteries, Electrochemical processes, Ionic transport, Electronic migration, Dipoles

## Abstract

Achieving high-energy density remains a key objective for advanced energy storage systems. However, challenges, such as poor cathode conductivity, anode dendrite formation, polysulfide shuttling, and electrolyte degradation, continue to limit performance and stability. Molecular and ionic dipole interactions have emerged as an effective strategy to address these issues by regulating ionic transport, modulating solvation structures, optimizing interfacial chemistry, and enhancing charge transfer kinetics. These interactions also stabilize electrode interfaces, suppress side reactions, and mitigate anode corrosion, collectively improving the durability of high-energy batteries. A deeper understanding of these mechanisms is essential to guide the design of next-generation battery materials. Herein, this review summarizes the development, classification, and advantages of dipole interactions in high-energy batteries. The roles of dipoles, including facilitating ion transport, controlling solvation dynamics, stabilizing the electric double layer, optimizing solid electrolyte interphase and cathode–electrolyte interface layers, and inhibiting parasitic reactions—are comprehensively discussed. Finally, perspectives on future research directions are proposed to advance dipole-enabled strategies for high-performance energy storage. This review aims to provide insights into the rational design of dipole-interactive systems and promote the progress of electrochemical energy storage technologies.

## Highlights

- Offers a thorough review on the mechanism of molecular and ion dipoles in high-energy batteries, covering development, classification, and multifaceted roles in battery systems.
- Elucidates how molecular and ion dipoles regulate ionic transport, optimize solvation structures, strengthen the electric double layer, and construct stable solid electrolyte interphase/cathode–electrolyte interface layers, all of which boost battery performance.
- Demonstrates the wide-ranging applications of dipole interactions in various battery systems, such as suppressing dendrites in lithium–metal batteries and improving the cycling stability of lithium–sulfur batteries.
- Proposes future research directions including AI-assisted materials design, in-depth mechanism exploration, multidisciplinary integration, database establishment, and promoting practical applications, aiming to drive the development of high-energy batteries.

## Graphical abstract

**Figure:**
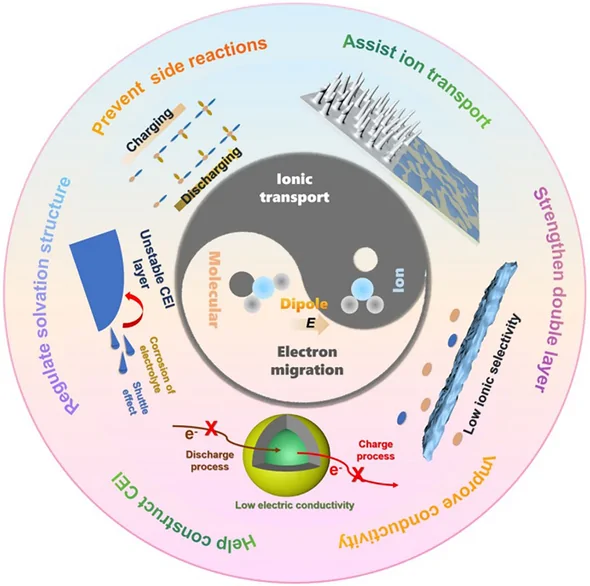

## Introduction

The escalating global demand for sustainable energy, coupled with the pressing challenges of environmental pollution and resource depletion, has placed unprecedented emphasis on the development of advanced energy storage technologies [1–4]. Among various candidates, high-energy density batteries have emerged as a cornerstone for powering diverse applications, ranging from portable electronics and electric vehicles to large-scale grid storage systems [5–8].

**Fig. 1:**
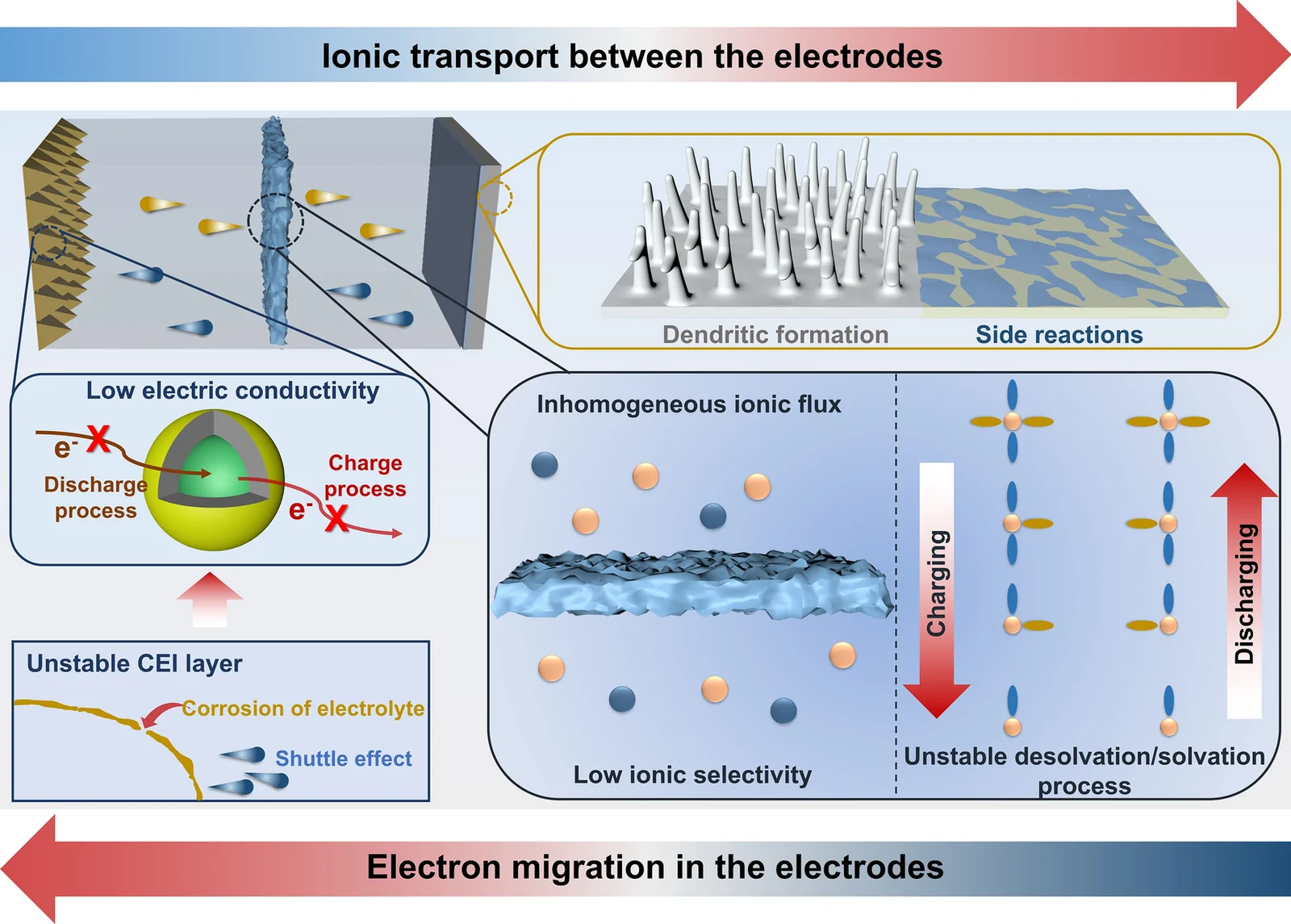
Critical issues in high-energy batteries related to ionic/electric transport

Achieving higher energy density is paramount for next-generation batteries, as it directly governs the amount of energy stored and delivered per unit weight or volume [8–10]. Central to this objective are the processes of ionic and electronic transport, which are intricately linked to nearly every component within the battery system, including cathodes, anodes, electrolytes, and separators (Fig. 1) [11–14]. However, several intrinsic and extrinsic limitations continue to impede progress. For instance, poor electronic conductivity in cathode materials restricts charge transfer kinetics during electrochemical reactions [15, 16], while instabilities at the cathode–electrolyte interface (CEI) exacerbate side reactions and structural degradation [17–19]. On the anode side, dendrite formation and surface corrosion, induced by inhomogeneous and undesired ionic transport, are prevalent and critical issues that not only undermine cycling stability, but also pose significant safety risks [20]. Additionally, high-energy systems, such as lithium–sulfur batteries, suffer from polysulfide shuttling, where the migration of soluble intermediates deteriorates active material utilization and compromises overall performance [21, 22]. Further complications arise from electrolyte failure, poor ion selectivity of separators, and unstable desolvation/solvation processes, collectively impairing the electrochemical stability and longevity of batteries [23–25]. Critically, these challenges are rooted in the complex interplay of ionic and electronic behaviors at the micro- and nano-scale interfaces and phases within the battery architecture. Addressing these fundamental transport issues from an interfacial and microstructural perspective offers a promising pathway toward the rational design of stable, high-energy density battery systems.

**Fig. 2:**
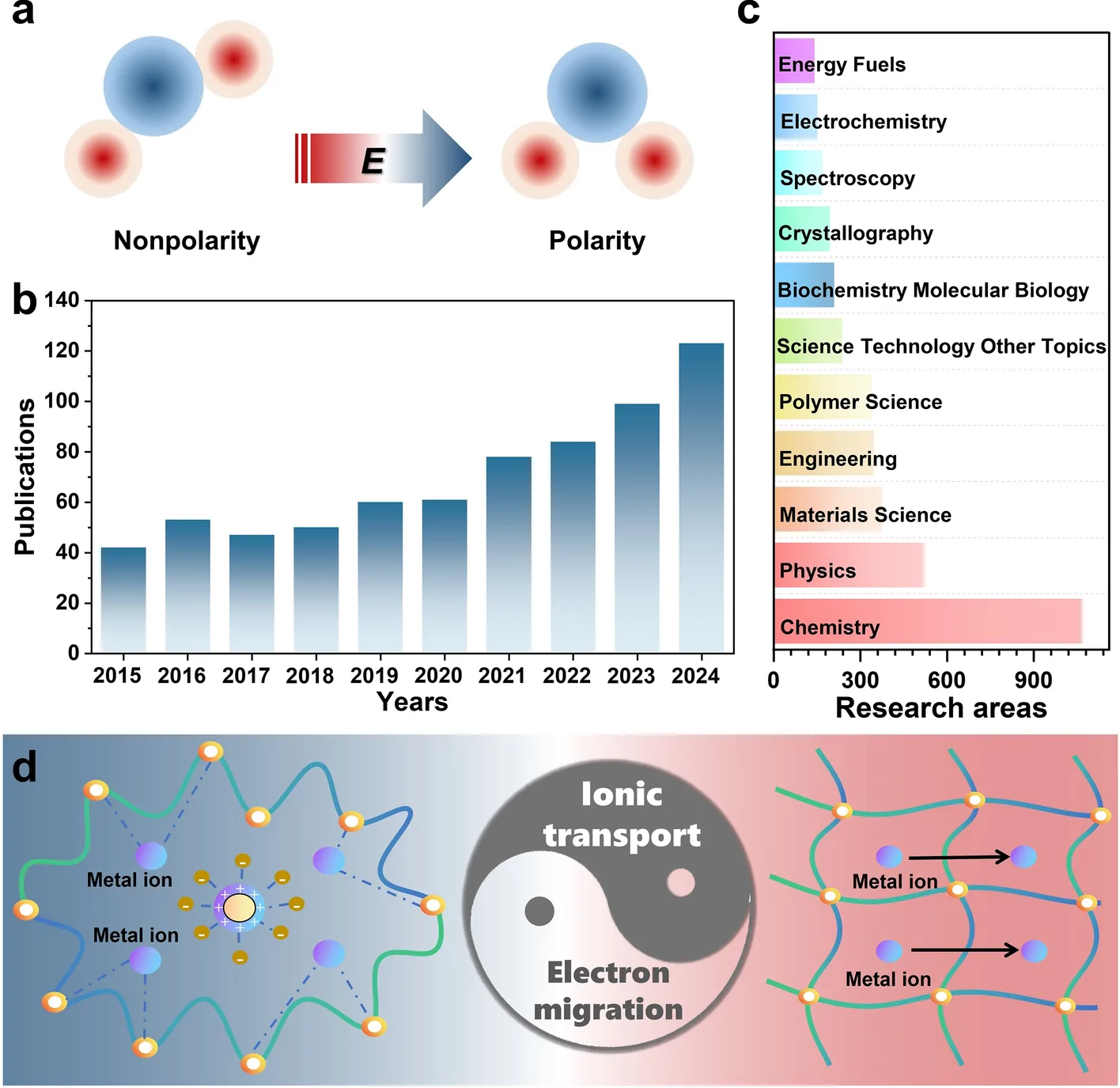
**a** Schematic of molecular and ionic dipoles. **b** Publishes and **c** research areas of dipoles (Data are from Web of Science). **d** Roles of molecular and ionic dipole in high-energy batteries

Dipole interactions between molecules and ions have recently emerged as a promising strategy to address these challenges, offering versatile and complex mechanisms with significant potential for performance enhancement [26–29]. These interactions originate from the uneven distribution of positive and negative charges within molecular or ionic species, resulting in strong electrostatic attractions or modifications at interfaces (Fig. 2a) [30, 31]. Such dipolar effects can effectively tailor the local physicochemical environment and modulate interfacial properties, thereby improving ionic and electronic transport processes [32–35]. For example, it could regulate the ionic transport process by optimizing the solvation structure of ions, facilitating the smooth movement of the ions between the electrodes and enhancing the transfer efficiency [36–38]. On the cathode side, they contribute to increased ionic conductivity and facilitate the formation of robust and uniform CEI layers, thus stabilizing the interface and promoting active material utilization [39–43]. Similarly, at the anode, dipoles can adjust the electric double-layer and solvation structure to stabilize the solid electrolyte interphase (SEI), suppress side reactions, mitigate dendrite formation, and maintain structural integrity [44, 45]. Furthermore, it could also contribute to reducing the shuttle effect of polysulfides and strengthening the stability of the electrolyte by forming a protective layer or modifying the interfacial chemistry [28, 46]. These functionalities underscore the critical role of dipole interactions in enabling high-performance, high-energy density batteries. Despite some impressive reviews concerning dipoles, including regulating ion transport and desolvation [47], regulating solvated shell [48, 49], interfacial stability, and other mechanisms [50], none of them comprehensively explore their multifunctional roles. Therefore, a systematic understanding of these mechanisms is urgently needed to guide the rational design of advanced battery systems with enhanced electrochemical performance.

In this review, we present a comprehensive overview of recent advances in dipole interactions and their emerging roles in enhancing the electrochemical performance of high-energy batteries. We begin by discussing the development, classification, and inherent advantages of dipole interactions in electrochemical systems. Next, we detail the fundamental mechanisms through which molecular and ionic dipoles regulate ionic transport, optimize solvation structures, modulate electric double layers, stabilize SEI and CEI, enhance electronic conductivity, and suppress side reactions and corrosion. Finally, we provide an outlook on future research directions to further harness dipole interactions for next-generation high-energy battery technologies.

## Development, Advantages, Classification, and Challenges in the Application of Dipole Interactions in High-Energy Batteries

### Development of Dipole Interactions in High-Energy Batteries

Molecular and ion–dipole interaction is a chemical phenomenon based on electrical interactions, which occurs between ions and molecules possessing dipole moments [51]. The dipole moment of a molecule originates from differences in electronegativity between constituent atoms, leading to an asymmetric electron cloud distribution [52]. This causes one end of the molecule to be positively charged and the other end to be negatively charged, thus forming a polar state similar to that of a "tiny magnet." When an ion approaches such a molecule, it interacts with the positively or negatively charged end of the molecule through electrostatic attraction, and this interaction is defined as the molecular ion–dipole interaction.

In recent years, extensive efforts have been devoted to harnessing these interactions to regulate ionic and electronic transport in various high-energy battery systems, including lithium-ion, sodium-ion, and lithium–sulfur batteries [53–57]. This research field has witnessed rapid growth, as evidenced by the increasing number of publications indexed in major scientific databases (Fig. 2b). A bibliometric analysis reveals a steady year-on-year rise in scholarly articles focusing on dipole interactions in batteries, with an expanding presence in top-tier journals across energy, chemistry, and materials science disciplines (Fig. 2c). These developments reflect the growing recognition of the multifunctional roles that molecular and ionic dipole interactions could play at the micro- and nano-scales within batteries, which mainly include two forms (Fig. 2d). Specifically, these interactions have been demonstrated to not only modulate solvation structures and ionic transport, but also contribute to interfacial stabilization, dendrite suppression, and improved active material utilization. Their inherent versatility provides an emerging platform for addressing persistent challenges in achieving both high-energy density and operational stability. Despite these promising advances, the systematic understanding and application of dipole interactions in battery systems remain at an early stage. Continued investigation is necessary to deepen mechanistic insights and unlock their full potential for next-generation high-energy battery technologies.

### Advantages of Dipole Interactions

Compared to conventional ion–ion electrostatic interactions, molecular ion–dipole interactions offer greater precision in modulating ion transport behaviors [47]. Unlike other chemical bonding mechanisms, such as coordination or covalent bonding, dipole interactions exhibit distinctive functionalities at electrode–electrolyte interfaces, where they can dynamically adjust the local ionic environment without introducing undesirable side reactions [50]. During battery operation, the electrode–electrolyte interface plays a pivotal role in governing electrochemical performance and stability. Many chemical interactions at this interface can result in the formation of unstable intermediates or trigger parasitic reactions, compromising battery longevity. In contrast, dipole interactions provide a balanced interaction strength—stronger than van der Waals forces yet milder than conventional covalent bonds—allowing for adaptive interface regulation, while minimizing the risk of irreversible chemical transformations. Moreover, in comparison with specific chemical interactions, such as the selective coordination between certain metal ions and ligands, dipole interactions display superior universality and material compatibility, enabling their application across a broad range of electrode and electrolyte chemistries [34]. These intrinsic advantages position dipole interactions as a versatile and scalable strategy for enhancing the performance and durability of high-energy batteries.

### Classification of Dipole Interactions in High-Energy Batteries

Dipole interactions in high-energy battery systems can be broadly categorized into the following types (Table 1): i) Ion–solvent molecule dipole interactions, where solvent dipoles modulate the solvation environment of ions, directly impacting ion transport, desolvation, and interfacial behaviors [41, 58, 59]. (ii) Ion–functional group dipole interactions, occurring at electrode surfaces modified with polar functional groups, which tailor interfacial charge distribution and influence ion flux dynamics [60]. (iii) Additive molecule ion–dipole interactions, where dipolar additives in electrolytes or separators interact with migrating ions, facilitating interfacial stabilization or selective ion transport [61]. These categories reflect the diverse contexts in which dipole interactions have been effectively integrated into various battery systems, including lithium-ion, sodium-ion, and lithium–sulfur batteries. Tailored dipole design strategies have demonstrated the ability to significantly improve electrochemical performance metrics, such as cycling stability, Coulombic efficiency, and rate capability. Given their structural diversity and functional tunability, dipole interactions offer a multifaceted toolbox for addressing multiple challenges simultaneously in high-energy battery systems. Continued exploration of these interaction types will further enhance design strategies for advanced battery materials and interfaces.

**Table 1:** The classification of some typical applications of dipole interactions in battery systems

Battery system	Dipole mechanism of action	Active substance	Applications	Refs
Lithium–metal batteries	Ion–solvent molecule dipole	PBM-IL-Li	Electrolyte	[58]
Sodium ion batteries	Ion–solvent molecule dipole	2-methyltetrahydrofuran	Electrolyte	[59]
Lithium–metal batteries	Ion–dipole action of functional groups on the surface of the electrode	WSEs	Electrolyte	[60]
Lithium–metal batteries	Additive Molecules Ionic Dipole Interaction	Methyl pyrrolidone	Electrolyte	
Lithium batteries	Ion–solvent molecule dipole	Difluoro (oxalato) borate	Electrolyte	[61]
Lithium–metal batteries	Ion–dipole action of functional groups on the surface of the electrode	Pyrrolidone	Electrolyte	[45]

## Mechanism of Molecular Ion Dipoles for High-Energy Batteries

**Fig. 3:**
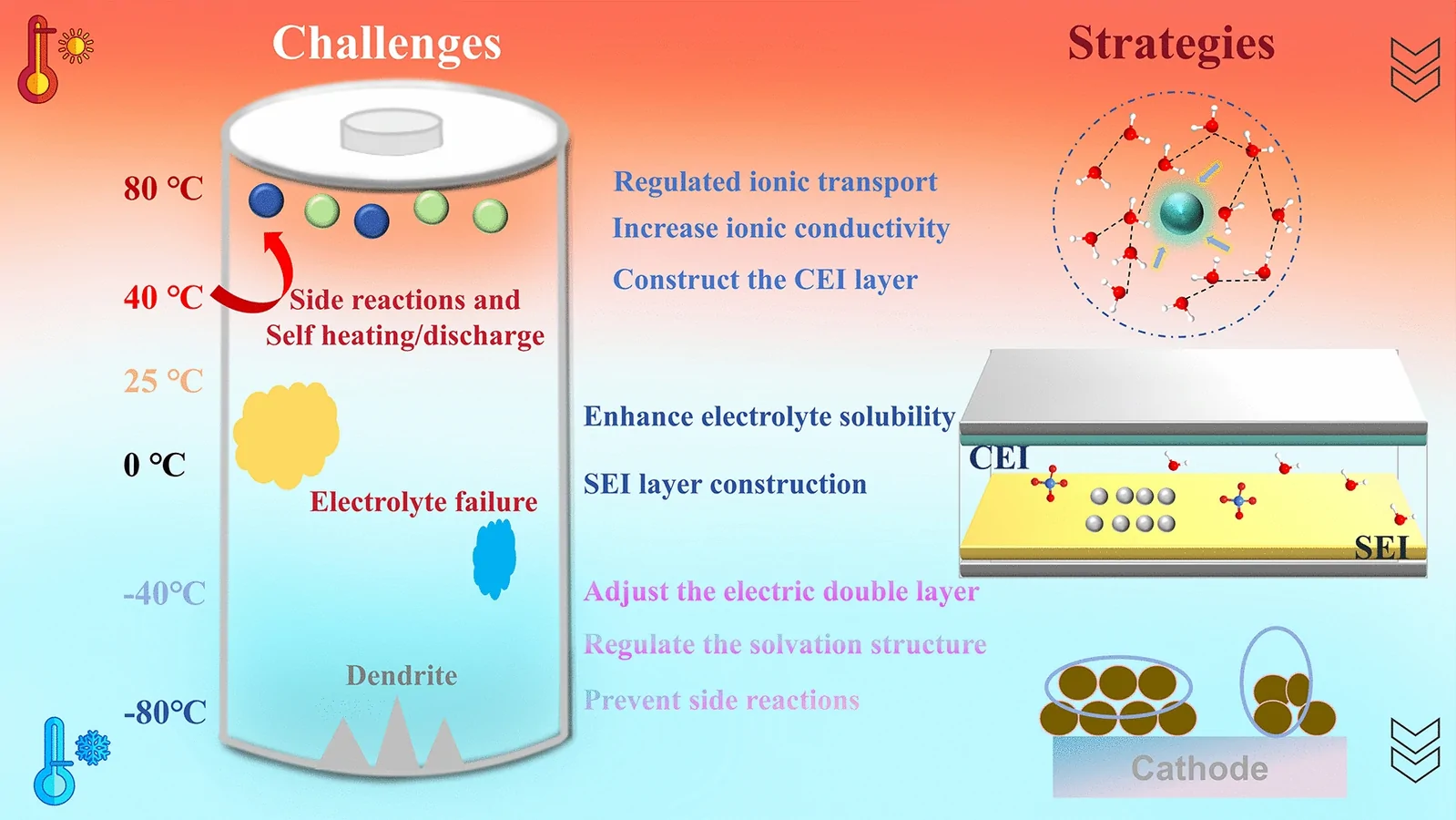
The role of molecular ion–dipole action in high-energy batteries

Based on the special advantages of molecular ion coupling, molecular ion coupling has been proved to be a promising candidate for solving the ionic electronic transport related issues of high-energy batteries [62, 63]. According to recent studies, the corresponding mechanism of molecular ion–dipole interaction in high-energy batteries could be classified concerning regulating the ionic transfer process, regulating and intervening in the desolvation process, enhancing the electric double layer, optimizing the structure of the SEI layer, improving the CEI layer, and elevating the electrical conductivity and so forth [44], as illustrated in Fig. 3. Table 2 summarizes the ionic transfer number, ionic conductivity, and some typical electrochemical performances including cyclic performance, plating/stripping stability, and Coulombic efficiency of some high-energy batteries with different molecular ion dipole effects.

**Table 2:** The ionic transfer number, ionic conductivity, and some typical electrochemical performances including cyclic performance, plating/stripping stability, and Coulombic efficiency of some high-energy batteries with different molecular ion–dipole effects

Materials	Ionic transference number	Ionic conductivity	Cyclic performance	Plating /stripping stability	Coulombic efficiency	Refs
PHMP	–	1.26 × 10^−4^ S cm^−1^	81% after 250 cycles under 0.2C	Over 1600 h at 1 mA cm^−2^/1 mAh cm^−2^	99.8% at 1.0 mA cm^−2^	[64]
(PVDF) − PbZr_x_Ti_1−x_O_3_ (PZT) CPE	0.37	1.16 × 10^−4^ S cm^−1^	86.2% after 500 cycles under 0.5C	Over 1900 h at 0.1 mA cm^−2^/0.1 mAh cm^−2^	–	[65]
CPEs	–	6.2 × 10^−5^ S cm^−1^	94% after 350 cycles under 1C	Over 1400 h at 0.2 mA cm^−2^/0.2 mAh cm^−2^	–	[66]
PEO-LiTFSI-NBS	0.77	1.08 × 10^−4^ S cm^−1^	–	Over 500 h under 25 μA cm^−2^	99.92% at 50 μA cm^−2^	[30]
NT-B	–	3.98 × 10^−5^ S m^−1^	75% after 2500 cycles under 0.5 A g^−1^	–	–	[67]
NG3F	–	–	80% after 170 cycles under 0.3C	–	98.62% at 0.5 mA cm^−2^/1mAh cm^−2^	[68]
CLSPE-IL	–	2.77 × 10–4 S cm^−1^	91% after 500 cycles under 0.2C	Over 2000 h 0.1 mA cm^−2^, 0.2 mAh cm^−2^	99.49% at 0.1C	[39]
PDMAPS	0.76	12.54 mS^−1^	86.4% after 2000 cycles under 3C	Over 9000 h under 0.5 mA cm^−2^/0.5mAh cm^−2^	84.1% at 1C	[69]
PCS-VCF/Li	–	–	62.5% after 500 cycles under 5C	Over 1500 h under 20 mA cm^−2^	97.0% at 1.0 mA cm^−2^, 1.0mAh cm^−2^	[44]
NDCPE-5%	0.75	0.29 mS cm^−1^	80.0% after 300 cycles under 1C	Over 400 h 0.5 mA cm^−2^/0.5 mAh cm^−2^	–	[70]
a-PE	0.74	5.1 × 10^−4^ S cm^−1^	96.4% after 1000 cycles under 5C	Over 900 h 0.1 mA cm^−2^/0.1 mAh cm^−2^	97.8 at 0.1C (initial CE)	[71]

### Boosting Ionic Transport

Dendritic formation has great impact on the electrochemical performance of high-energy batteries [70–73]. The formation of dendrites is essentially the non-uniform deposition caused by the uneven transport of metal ions. Therefore, it is of great importance to suppress the formation of dendrites. The dipole effect can regulate the transport process of metal ions, adjust the surface electric field and ion distribution, form ordered ionic channels, accelerate the migration of metal ions, and increase the transference number of metal ions, thereby enabling dendrite-free metal deposition [30, 67, 68, 70].

#### Promoting Uniform Ionic Transport

**Fig. 4:**
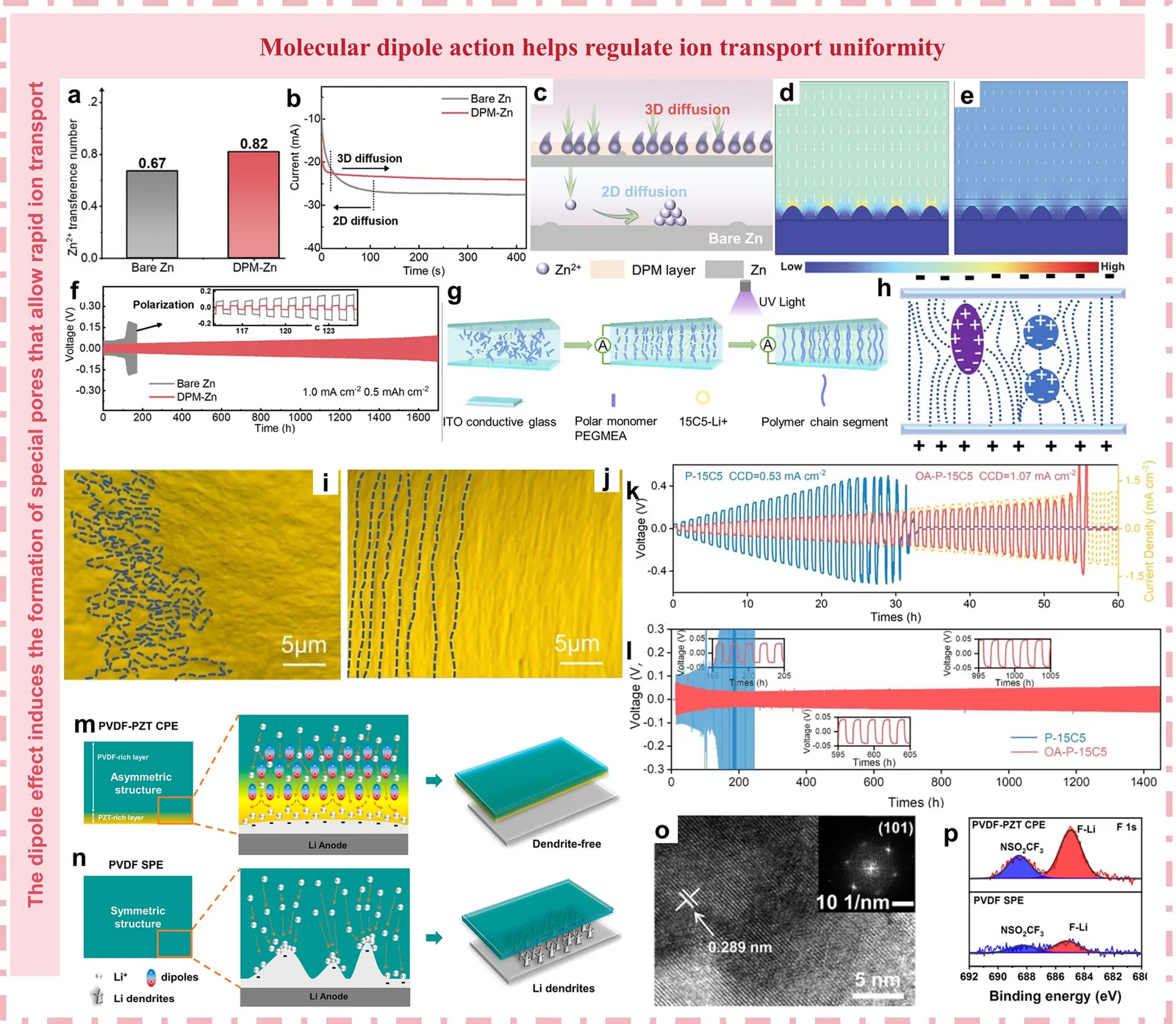
**a** Calculated Zn^2+^ transfer number. **b** Chronoamperometric curves at −150 mV. **c** Schematic diagram of the diffusion behavior of zinc ions on different electrode surfaces. COMSOL simulations of electric field distribution of **d** bare Zn and **e** DPM-Zn. **f** Galvanostatic cycling performance of bare Zn and DPM-Zn symmetric cells at 1 mA cm^−2^, 0.5 mAh cm^−2^. Preparation of OA-P-15C5 composite polymer electrolytes. Reproduced with permission from Ref. [54]. Copyright 2024 Wiley–VCH GmbH. **g** A schematic diagram of the electric field-induced molecular alignment. **h** Diagram of polar particles’ electric field driving effect. Light microscope photographs of **i** P-15C5 CPEs and **j** OA-P-15C5 CPEs. **k** Galvanostatic Li plating/stripping profiles of the symmetrical Li|P-15C5|Li cells and Li|OA-P-15C5|Li cells at step-increased current densities. **l** Galvanostatic cycling of symmetric Li|P-15C5|Li cells and Li|OA-P-15C5|Li cells at 0.2 mA cm^−2^ and 0.2 mAh cm^−2^. Reproduced with permission from Ref. [66]. Copyright 2024 Elsevier B.V. Schematic of **m** uniform Li^+^ flux guided by the dipolar channels of PZT/PVDF − PZT CPE interfaces near the Li anode and **n** disordered Li^+^ flux in the PVDF SPE that induces lithium dendrite growth, the inserts show the partially enlarged images with different cycles. **o** HRTEM image of PZT. The inset is the corresponding SAED pattern. **p** F 1* s*, XPS spectra of cycled Li anodes from Li/PVDF − PZT CPE/Li and Li/PVDF SPE/Li symmetrical cells. Reproduced with permission from Ref. [65]. Copyright 2023, American Chemical Society

The ionic transport in an ordered and oriented structure is much faster than that in a randomly arranged one, which could maximize the ion migration and thus greatly improve the corresponding performance [47, 74–76]. Cai et al. proposed a regulation of ion field for the zinc anode by the dipole molecular effect and constructed a unique dipole molecular (DPM) layer on the zinc surface to adjust the surface electric field and ion distribution. It was also verified that the construction of ordered ion channels through the dipole effect enabled the rapid migration of zinc ions [54]. As shown from Fig. 4a, the dipole effect exhibited rapid zinc ion migration kinetics. DPM-Zn had a relatively high Zn^2+^ transference number of 0.82, which was higher than that of the bare zinc electrode (0.67), indicating its outstanding ion transport ability. The diffusion and nucleation behavior of zinc ions on the surface of DPM-Zn was further investigated by chronoamperometry (CA). The rapid decrease in current in the initial stage indicated the diffusion and nucleation of zinc on the electrode surface, and the subsequent stable current represented its growth process [77]. As shown in Fig. 4b, at a constant voltage of −150 mV, the CA curve of bare zinc exhibited a continuously decreasing current density over 100 s, suggesting a long-term two-dimensional (2D) diffusion. Because zinc ions were more inclined to diffuse laterally toward the tip position, and the accumulation of excess charges at the tip position was more conducive to deposition. Figure 4b shows that, compared with the bare zinc electrode, the zinc surface with the DPM layer could effectively promote the rapid nucleation and growth of zinc on the electrode surface. In addition, the DPM layer improved the uniform distribution of ions on its surface, induced the uniform deposition of zinc ions, accelerated the interfacial migration of ions, and was beneficial for improving the electrochemical performance of zinc-ion batteries [78, 79]. The bare zinc surface was rough with tiny protrusions (Fig. 4c).

In addition, these protrusions exhibited a relatively strong local electric field, leading to charge accumulation and attracting more Zn^2+^, resulting in the rapid growth of Zn dendrites (Fig. 4d). The formation of zinc dendrites is due to the non-uniform deposition of zinc ions in aqueous electrolytes, which mainly based on non-uniform nucleation and non-uniform electric field induction. After the introduction of the DPM layer, the DPM-Zn electrode exhibited a uniform and reduced interfacial electric field (Fig. 4e), mainly because the DPM had a unique local dipole structure and could form an electric dipole layer on the interface through the dipole effect to regulate the charge distribution, thus homogenizing the spatial electric field on the electrode–electrolyte interface. The surrounded hydrophobic groups could serve as a propeller for the accelerated movement of zinc ions and accelerate the ion migration by dipole repulsion [80]. This unique structure was evenly distributed throughout the DPM layer and forms a transport channel for zinc ions. These results indicated that the DPM with intramolecular dipoles could eliminate the tip effect by homogenizing the interfacial electric field, deposited zinc uniformly, and accelerated the migration of zinc ions. It was precisely because of this that the zinc-ion symmetric battery was able to cycle stably for more than 1700 h at a current density of 1 mA cm^−2^, while maintaining a lower polarization voltage (Fig. 4f). Compared with the bare zinc electrode, the polarization voltage of the latter suddenly increased only after running for 100 h [81]. The reduced voltage hysteresis and prolonged cycle life of DPM-Zn indicated that the introduction of the DPM layer reduced the resistance of the zinc ion interfacial migration and improved the reversibility of the plating stripping [82]. This study provided an idea for the dipole effect to induce ions in regulated channels and promoted ionic transport.

The application of solid-state lithium–metal batteries (LMBs) requires the preparation of flexible composite polymer electrolytes (CPEs) with vertically aligned ion transport paths. However, the orientation of the polymer matrix is usually disordered, so the ion transport process is not necessarily uniform. Similar to the research of Cai et al., Zhao et al. also developed a novel and simple electric field induced molecular targeting strategy for the fabrication of flexible CPEs with vertically aligned ion transport channels [66]. Motivated by a straight current (DC) electric field (such as 1.5 MV m^−1^), the polar 15C5-Li^+^ dipole solidified with the monomer molecules in a regular orientation and formed a flexible three-dimensional (3D) cross-linked structure through the dipole effect. As a result, many perpendicularly aligned ion transport channels were created in the vertically oriented CPEs, thus showed an excellent ability to suppress the growth of lithium dendrites and promote the ionic transport process. Figure 4g illustrates the preparation of the OA-P-15C5 composite polymer electrolyte with a vertically oriented molecular structure induced by in situ photo-initiated polymerization in a DC electric field. During the preparation process, both PEGMEA monomer and PEGDA crosslinker contained ethylene oxide polar units (–CH_2_–CH_2_–O–), which had good coordination with 15C5. At the same time, there was a strong coupling effect between the crown ether ring of 15C5 and Li^+^, which could accelerate the dissolution and dissociation of lithium salt (LiTFSI) and formed a large number of dipoles (15C5-Li^+^) [83]. When a strong electric field is applied, these polar dipoles could be further polarized and regularly arranged along the direction of the electric field, thus driving the polar molecules of PEGMEA and PEGDA to reorient in this strong electric field (Fig. 4h). As a result, these regularly oriented molecular chains that contain unsaturated acrylic groups were readily polymerized and fixed by photoinitiator-184 under UV light to form three-dimensional (3D) cross-linked structures with vertically aligned ionic transport channels. In addition, both PEGMEA and 15C5 were readily polarized in the presence of a DC electric field and were uniformly distributed along the direction of the electric field. Obviously, this unique orientation was pretty beneficial to the transport of lithium ions. In order to clearly understand the microstructural differences of the CPEs prepared under the orientation induced by a DC electric field, their cross-sectional morphologies were observed by an optical microscope. As shown in Fig. 4i, the surface of P-15C5 had no regular orientation stripes, indicating that the structure of P-15C5 was randomly arranged. Interestingly, when an external DC electric field (for example, 1.5 mV m^−1^) was applied before the photopolymerization of the precursor solution, this electric field could induce the 15C5-Li^+^ and PEGMEA molecules to be arranged parallel to the direction of the electric field and formed a three-dimensional network of interconnected channels after photopolymerization. Therefore, the cross-section of OA-P-15C5 CPE showed a large number of slightly striped patterns in the parallel direction, as shown in Fig. 4j. This regular arrangement was driven by the dipole interaction caused by the strong DC electric field. For this reason, the vertically oriented OA-P-15C5 CPEs provided a critical current density (CCD) of 1.07 mA cm^−2^, which was higher than that of the randomly arranged P-15C5 CPEs (0.53 mA cm^−2^), indicating an enhanced ability to suppress the growth of lithium dendrites (Fig. 4k). Similarly, Fig. 4l demonstrates that the symmetrical Li||Li cell assembled with vertically oriented OA-P-15C5CPEs operated stably for over 1400 h at a current density of 0.2 mA cm^−2^ without significant voltage fluctuations. In contrast, the symmetrical Li||Li cell assembled with randomly arranged P-15C5 CPE failed to maintain stability even after 200 h of cycling, which revealed the ultrastable stripping/plating behavior at the interface between OA-P-15C5CPEs and the lithium–metal anode (LMA). The improved suppression of lithium dendrites could be attributed to faster ion transport and weaker polarization in the charge–discharge cycle, and ultimately, these results were brought by the formation of ordered and oriented channels through the dipole effect to induce the electric field and regulate the charge distribution.

Different from the previous studies, Huang et al. prepared an asymmetric poly (vinylidene fluoride) (PVDF)-PbZr_x_Ti_1-x_O_3_ (PZT) CPE [65]. The CPE combined high dielectric PZT nanoparticles and enriched the dense thin layer on the anode side, making the ends of its dipoles highly electronegative. These dipoles with high electronegativity attracted Li^+^ on the PVDF–PZT interface to transport through the dipole channels and promoted the dissociation of lithium salts into free Li^+^. The PVDF-PZT CPE had an integrated structure, and its asymmetric composition was naturally formed due to the precipitation of heavy PZT in a dilute solution. More importantly, the positions of the central Zr or Ti atoms relative to the O atoms at the corners of PZT change under the electric field [84, 85], which generated a large number of dipoles with opposite electronegativities. These dipoles attracted Li^+^ on the PZT − PVDF interface, guiding the transport of Li^+^ between adjacent dipoles, and effectively constructed dipole channels (Fig. 4m, n). In addition, the high-resolution transmission electron microscopy (HRTEM) images and selected area electron diffraction (SAED) pattern analysis of PZT showed that its lattice spacing was 0.289 nm (Fig. 4o), consistent with the (101) plane of tetragonal PZT. The tetragonal phase was a ferroelectric phase, endowing PZT with a high dielectric constant [86], promoting the dissociation of salts, generating more electronegative dipole ends, and enabling Li^+^ to be adsorbed and transported along the dipole channels. Moreover, the pure tetragonal phase, different from the mixed tetragonal and rhombohedral phases that provided dipoles in different directions, ensured the alignment of dipoles in a single direction, thus explaining the mechanism of the dipole effect.

The cycled Li//PVDF − PZT CPE interface showed more lithium fluoride than the cycled Li//PVDF SPE interface (Fig. 4p). This could be attributed to the presence of PZT near the Li metal surface, which provided a strong dipole moment, accelerated the degradation kinetics of the C-F bond cleavage in LiTFSI, and thus successfully formed a highly stable SEI film rich in LiF [87]. The formation of abundant lithium fluoride promoted the rapid transfer of Li^+^ and suppressed the growth of lithium dendrites.

#### Improve the Solubility of the Electrolytes

As a key component of batteries, the electrolytes directly affect the overall performance and service life of batteries with its physical and chemical properties. Typical problems, such as electrolyte solubility and electrolyte failure in different environments, also exist in the electrolyte. The nature of these problems could be partly attributed to ionic transport problems [88, 89]. The molecular ion dipole interaction plays an indispensable role in optimizing the characteristics of the electrolytes and has become one of the hotspots in the field of battery research in recent years [90–93]. Numerous studies have shown that it makes significant contributions to improve multiple physical and chemical properties of the electrolytes. Taking the solubility of the electrolytes as an example, appropriate solvent molecules, by virtue of their dipole characteristics, could generate mutual attraction with the anions and cations of lithium salts, and provide a more favorable dissolution environment for lithium salts and increase the solubility of lithium salts in the electrolytes.

**Fig. 5:**
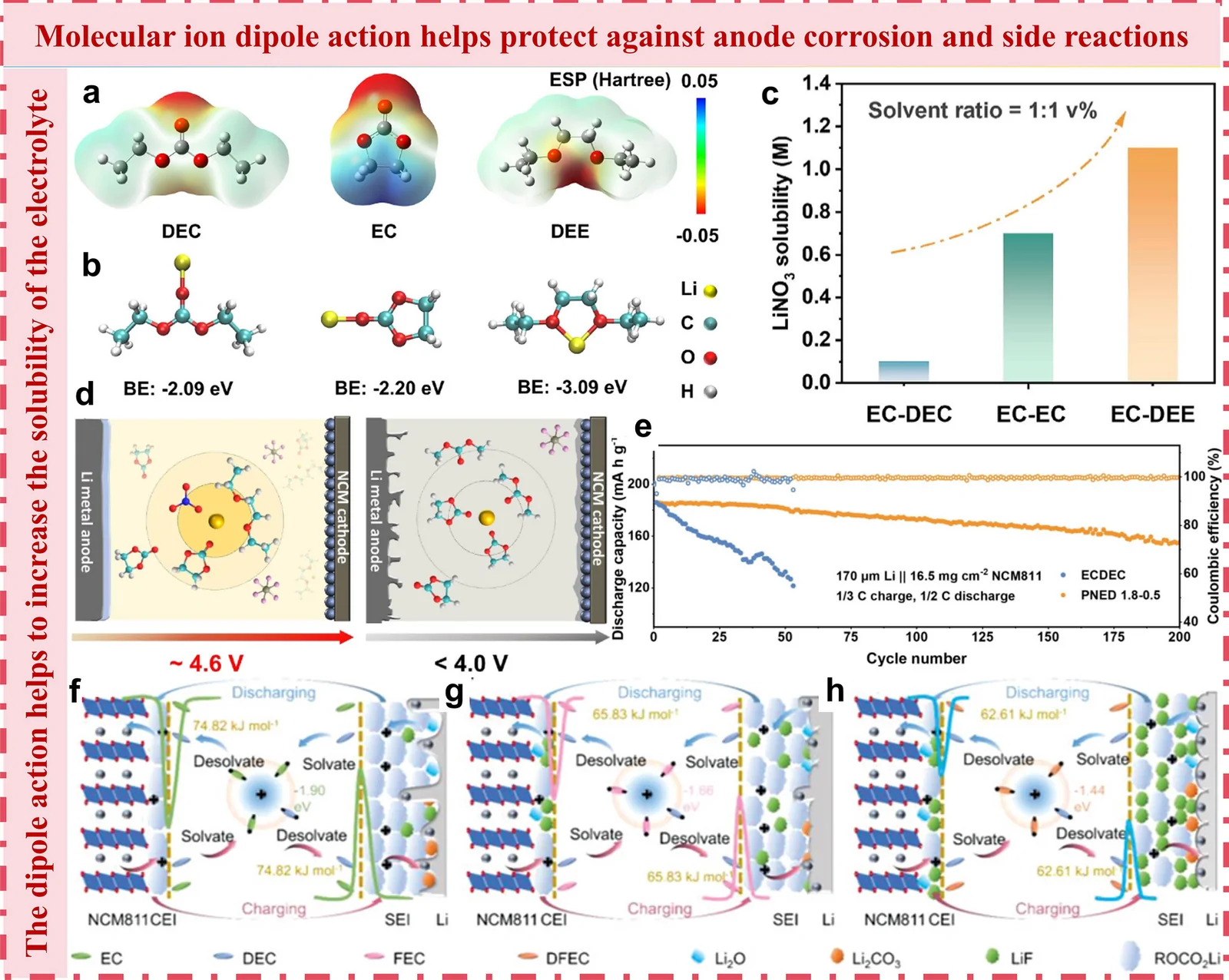
**a** Electrostatic potential landscapes of solvent molecules. **b** Binding energies between Li^+^ and solvent molecules. **c** Solubility of LiNO_3_ in different systems. **d** Schematic representation of the solvated structures and function of different electrolytes. ECDEC on top, PNED 1.8–0.5 on bottom. **e** Cycling stability of Li||NCM811 cells at cut-off voltages of 4.3 V. Reproduced with permission from Ref. [94]. Copyright 2024 Science Press and Dalian Institute of Chemical Physics, Chinese Academy of Sciences. Schematics of the dynamic evolution of Li^+^ solvation sheath during charging/discharging processes in **f** 1 M LiPF_6_-EC/DEC, **g** 1 M LiPF_6_-FEC/DEC, and **h** 1 M LiPF_6_-DFEC/DEC electrolytes, respectively. Reproduced with permission from Ref. [95]. Copyright 2021 Wiley–VCH GmbH

Taking into account the advantages in this regard, Xu et al. successfully enhanced the solubility of LiNO_3_ through the meticulous regulation of ion–dipole interactions, thereby achieving compatibility with the lithium–metal anode and ensuring the stability of the high-voltage cathode [94]. The electrostatic potential (ESP) mapping and density functional theory (DFT) calculations were, respectively, employed to precisely reveal the atomic charge densities of diethyl carbonate (DEC), ethylene carbonate (EC), and diethyl ether (DEE), along with their binding energies in relation to Li^+^ (Fig. 5a, b). Among these compounds, the two oxygen atoms within DEE exhibited robust polarity, endowing them with the capacity to engage in double ion–dipole interactions with Li^+^. Consequently, DEE demonstrated a notably higher binding energy (−3.09 eV) compared to Li^+^-DEC (−2.09 eV) and Li^+^-EC (−2.20 eV). The reinforcement of ion–dipole interactions within the electrolyte played a pivotal role in facilitating the dissociation of lithium salts. In the solubility tests of LiNO_3_, the EC-DEC, EC-EC, and EC-DEE solvent systems (with a volume ratio of 1:1) were capable of dissolving 0.1, 0.7, and 1.1 M of LiNO_3_, respectively (as shown in Fig. 5c). When LiPF_6_ was utilized as the principal salt, LiNO_3_ proved to be rather difficult to dissolve in the 1.0 M LiPF_6_/EC-DEC electrolyte. However, the dissolution of 1.0 M LiPF_6_-0.3 M LiNO_3_/EC and 1.8 M LiPF_6_-0.5 M LiNO_3_/EC-DEE electrolytes could be effectively realized. As a result, the electrolytes designed in this study exhibited superior interfacial stability in comparison with traditional ester-based electrolytes (Fig. 5d), which significantly contributed to the improvement of the electrochemical performance of high-voltage LMBs. Figure 5e presents the cycling performance subsequent to cell activation. Specifically, the specific capacity of the cells employing the EC-DEC electrolytes witnessed a rapid decline to 121.7 mAh g^−1^ after merely 53 cycles, accompanied by a rather low capacity retention rate of merely 65.4%. In stark contrast, the Li||NCM811 cells utilizing the 1.8–0.5 electrolytes of poly(ethylene glycol) diacrylate (PNED) demonstrated an outstanding capacity retention rate of 83.6% even after 200 cycles, along with an average CE surpassing 99.6%.These experimental data unambiguously suggested that the introduction of DEE into the ester-based electrolytes enabled the construction of a solvation structure incorporating nitrate- and PF_6_-, which not only augmented the wettability, but also enhanced the solubility of the electrolytes. This, in turn, proved to be highly advantageous for stabilizing the lithium anode and elevating the electrochemical performance of high-voltage LMBs.

The enhanced performance could be ascributed into the follow reasons: Leveraging the substantial ion–dipole interaction between Li^+^ and DEE in the cell, the solubility of LiNO_3_ was remarkably enhanced. Moreover, the steric effect of DEE facilitated the participation of anions in the solvation structure, thereby effectively promoting the formation of an inorganic-rich SEI. Simultaneously, the low-viscosity characteristic of DEE also served to improve the interfacial wettability of the ester-based electrolyte. Thus, the designed electrolyte had significantly enhanced the electrodeposition morphology of the lithium anode, the Coulombic efficiency, and the cycling stability.

In contrast with the approach adopted by Xu et al., Wang et al. improved the low-temperature Li^+^ desolvation kinetics through the design of a fluorination-regulated Li^+^–dipole interaction to enhance the overall performance of the batteries [95]. This strategic modification led to an increased solubility of ions within the electrolyte, thereby further enhancing the overall performance of the battery. During the charging process, specifically in the context of the Li^+^ desolvation step of the Li^+^-based electrolyte, the DEC solvent, which exhibited a relatively low Li^+^–dipole interaction strength, is initially removed from the Li^+^–dipole structure (Fig. 5f). However, its relatively low reduction potential posed an obstacle to its initial reduction for the formation of the SEI. In contrast, EC possessed a relatively high reduction potential and was thus capable of forming a protective SEI, which effectively prevented the continuous decomposition of the electrolyte. This phenomenon was consistent with the dominance of the SEI formed by EC reduction compounds on graphite, where the outer layer was predominantly composed of organic ROCO_2_Li. Due to the deep reduction of ROCO_2_Li, the inner layer became rich in inorganic substances, such as lithium oxide and Li_2_CO_3_. When Li^+^ was transported through the SEI, the highly resistive organic ROCO_2_Li impeded the diffusion of Li^+^, resulting in an uneven charge distribution and subsequently giving rise to the growth of dendritic Li [96]. Upon replacing EC with fluoroethylene carbonate (FEC), the FEC molecules, owing to their lower interaction strength compared to DEC, were preferentially stripped from the Li^+^–dipole structure, thereby generating a LiF-rich SEI film with a high reduction potential (as shown in Fig. 5g). By comparison, difluoroethylene carbonate (DFEC) was more readily desolvated from the Li^+^–dipole structure than other solvents within the Li^+^ solvation sheath (Fig. 5h), thereby augmenting the solubility of ions within the electrolyte and favorably contributing to the enhancement of the electrochemical performance of the battery.

#### Blocking the Shuttle Effect of Polysulfides

In the previous research, it was found that in solid-state electrolytes (SSEs), due to the formation of larger-sized Li_2_S particles [97], during the charging process with a cut-off voltage of 2.8 V. The discharge product Li_2_S was not completely dissociated during the charging. The cyclic accumulation of the residual Li_2_S led to the rapid failure of the lithium–sulfur batteries (LSBs) [98, 99]. Fundamentally, the uneven distribution of Li_2_S particles could be ascribed to the poor interaction between polar Li_2_S and non-polar carbon substrates. Jiang et al. embedded copper sulfide nanoparticles [100], which have high dipole–dipole interactions with Li_2_S, into the carbon matrix of the sulfur cathode as polar sites. In this way, they homogenized the deposition of Li_2_S and accelerated the conversion kinetics, thus effectively solving the problem.

**Fig. 6:**
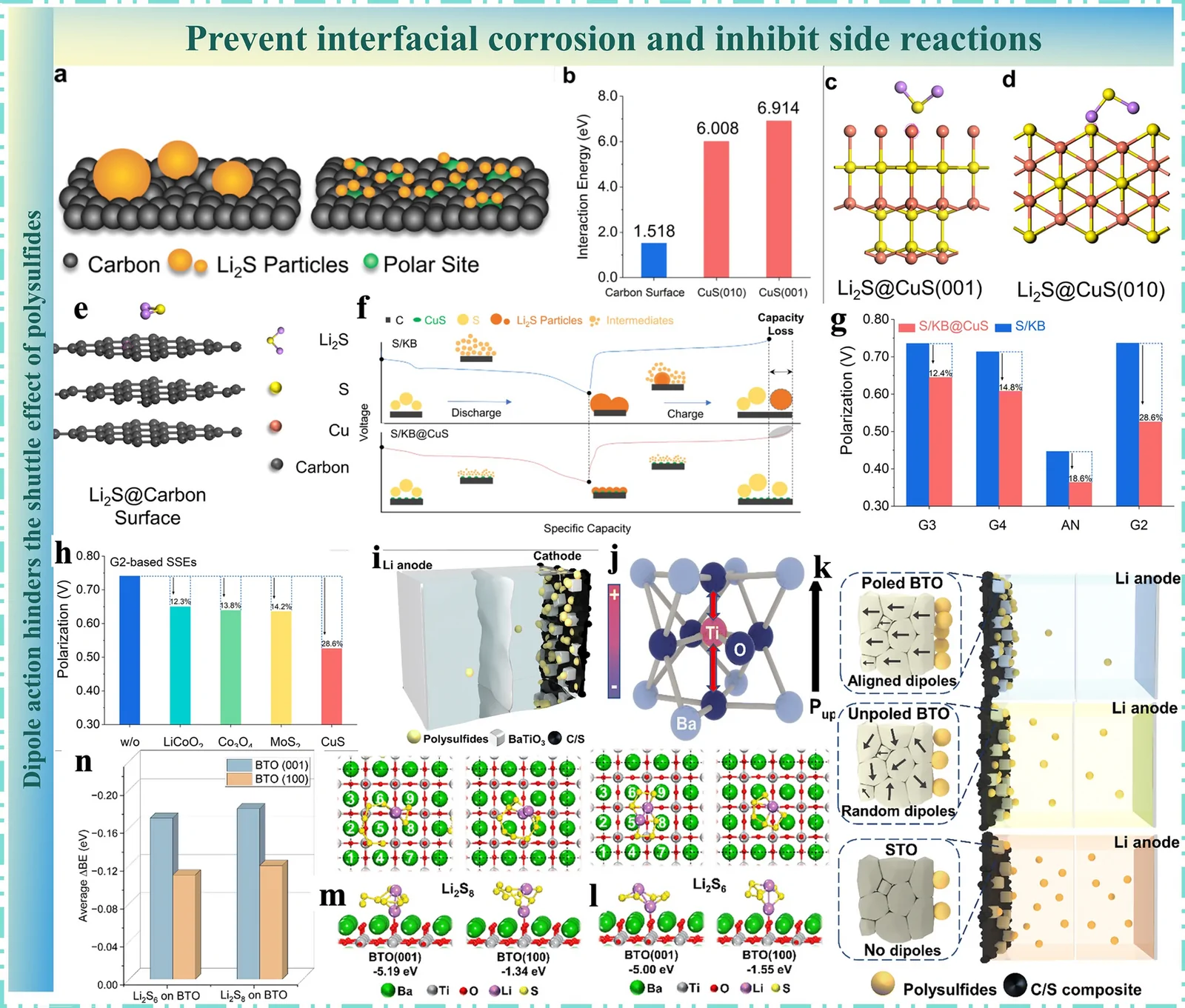
Schematics of the aggregation of Li_2_S in SSEs and molecular dynamics simulations of the interaction of Li_2_S with carbon and CuS. **a** Diagrams of the aggregation of Li_2_S at the surface of carbon and how the aggregation is inhibited with polar sites. **b** Adsorption energy and the corresponding situations of Li_2_S molecule adsorbed at the **c** (001) and **d** (010) planes of CuS, and **e** surface of the carbon. **f** Schematic showing the conversion of sulfur species during discharging/charging for the cells with S/KB (abov**e)** and S/KB@CuS (below) cathodes. The universality of the dipole–dipole interaction strategy. **g** Polarizations of the cells with S/KB@CuS and S/KB cathodes in AN-based, G3-based, G4-based, and G2-based SSEs. **h** Polarizations of the cells with S/KB@LiCoO_2_, S/KB@Co_3_O_4_, S/KB@MoS_2_, S/KB@CuS, and S/KB cathodes in G2-based SSE. Reproduced with permission from Ref. [100]. Copyright 2024 Elsevier B.V. Schematic illustrations of BaTiO_3_ nanoparticles in Li–S batteries. **i** A Li–S battery with a composite ferroelectric BaTiO_3_/C/S cathode. **j** A ferroelectric BTO lattice with upward polarization. **k** Polysulfide adsorption on cathodes of poled BTO, unpoled BTO, and non-ferroelectric STO. DFT calculations of the lowest-energy configurations of adsorption of **l** Li_2_S_6_ and **m** Li_2_S_8_ on BTO (001) and BTO (100) surfaces. **n** Simulated average Ba 3d binding energies of Li_2_S_6_ and Li_2_S_8_ on BTO (001) and (100). Reproduced with permission from Ref. [102]. Copyright The Royal Society of Chemistry 2024

To obtain smaller and uniformly distributed Li_2_S particles, one solution was to introduce polar sites. By generating dipole–dipole interactions between Li_2_S and the carbon matrix, more nucleation could be induced (Fig. 6a). Copper sulfide was chosen as the polar site on account of its unique sulfur affinity and electrical conductivity [101]. The results of molecular dynamics simulations demonstrated that the interaction energy between Li_2_S molecules and the (001) and (010) planes of copper sulfide was nearly five times greater than that between Li_2_S and the carbon surface (Fig. 6b–e). The chart in Fig. 6f illustrates the possible evolution process of sulfur species during the discharging/charging process. During the discharging process, compared with the S/KB cathode, Li_2_S tended to aggregate and form large particles. The polar copper sulfide nanoparticles provided sufficient nucleation centers at the cathode, resulting in the formation of small particles, thus enabling the uniform deposition of Li_2_S particles, shortening the transport paths of electrons and Li^+^, and facilitating the conversion of Li_2_S, avoiding the residue of Li_2_S during the charging process. The proposed dipole–dipole interaction strategy possessed relatively high universality. Firstly, it was verified by comparing the polarization of cells obtained from SSEs of acetonitrile (AN), triethylene glycol monomethyl ether (g3), tetraethylene glycol dimethyl ether (g4), and (g2). The results are shown in Fig. 6g. Among all the above SSEs, the cells with the S/KB@SCuS cathode had lower polarization. Moreover, as depicted in Fig. 6h, replacing copper sulfide with other polar materials such as LiCoO_2_, Co_3_O_4_, and molybdenum disulfide could also reduce the polarization rate. However, compared with copper sulfide, these other polar inorganic materials had weaker electrical conductivity and weaker interaction with Li_2_S. They did not improve the polarization of LSBs based on SSEs as effectively as copper sulfide did. In any case, this universality was beneficial for identifying other, more promising materials.

Unlike Zhang et al. who provided active sites to induce polysulfide shuttling, Jiang et al. proposed a new method using barium titanate (BTO) with controlled dipole arrangement as a ferroelectric additive to form highly aligned dipoles and generate a relatively strong electrostatic field for improving polysulfide capture [102]. In this work, they first explored a new approach of using a high-bipolar ferroelectric material, polar BTO, as a cathode additive for lithium-ion batteries (Fig. 6i). To study the influence of different degrees of dipole alignment, three cathodes, namely, polarized BTO, non-polarized BTO, and non-ferroelectric strontium titanate (STO), were prepared, and their performances were evaluated (Fig. 6k). In the tetragonal phase of BTO, the z-axis distortion of Ti atoms led to the non-overlap of charge centers and the self-polarization characteristic (Fig. 6j). This distortion did not exist in the non-ferroelectric STO. Therefore, the unique ferroelectric effect of BTO in Li–S batteries could be demonstrated by direct comparison. BTO and STO nanoparticles were synthesized by the molten salt method, and then, the electrodes were polarized using the corona polarization technique. Piezoelectric response force microscopy (PFM) was used to characterize the alignment of ferroelectric dipoles.

The adsorption of polysulfides on the BTO surface was further explored by DFT calculations for two BTO crystal planes: (001) and (100), which were perpendicular and parallel to the Ti distortion, respectively. The most stable adsorption complexes of Li_2_S_6_ and Li_2_S_8_ on BTO (001) and BTO (100) are shown in Fig. 6l, m. In both cases, the adsorption of polysulfides was an exothermic process, but it was more thermodynamically favorable on the polar surface (45.0 eV for BTO (001) and 1.3–1.6 eV for BTO (100)). Interestingly, the interaction between the adsorbate and the O atoms on the polar BTO (001) surface was relatively weak. For Li_2_S_6_ and Li_2_S_8_, the lithium bond lengths were 1.92 and 1.86, respectively, compared with 1.76 for the polysulfides on BTO (100). On the contrary, the adsorption strength of the two polysulfides on BTO (001) was stronger, probably due to the formation of more Ba-S contact points on BTO (001), which led to the shortening of the Ba-S bond distance to 3.20–3.60 Å, compared with 3.24–3.66 Å on the non-polar surface. The formation of these Ba-S contact points might be affected by the ferroelectric polarization along the (001) axis in the BTO unit cell. This observation was consistent with the DFT calculations, which also showed the average Ba displacement of BTO (001) (Fig. 6n). For each Ba atom in these four polysulfide–BTO adsorption complexes shown in Fig. 6l, m, the simulated Ba 3*d* peaks showed two sets of contributions. The lower binding energy environment corresponded to the interaction between the surface Ba atoms and the S atoms of Li_2_S_6_ or Li_2_S_8_ molecules, while the second set with higher binding energy corresponded to the Ba atoms that did not interact. These observations indicated that the polarized and anti-polarized BTO surfaces with highly aligned ferroelectric dipoles contributed to the formation of stronger Ba-S bonds, which was consistent with the DFT calculations and could effectively anchor the polysulfides on the cathode.

Therefore, the polarized BTO cathode not only exhibited superior initial capacity, but also had a lower concentration of shuttling polysulfides during the cycling process, resulting in the 24% increase in capacity after 500 cycles. Besides, the poled and reverse-poled BTO realized the highest first-cycle capacities of 1379 and 1368 mAh g^−1^ at C/10, while the unpoled BTO presented a first-cycle capacity of 1250 mAh g^−1^. The values were far higher than the initial capacities achieved in the poled STO (925 mAh g^−1^) and unpoled STO electrodes (862 mAh g^−1^). These improvements were attributed to the relatively strong electrostatic field caused by the highly aligned dipoles on the polarized BTO surface. These dipole interaction mechanisms laid a certain foundation for solving interface corrosion and suppressing the occurrence of side reactions and broadened the ideas for solving the problem of polysulfide shuttling.

### Assisting Solvation Behavior and Interferes with Solvation Structure

From the perspective of molecular theory, the metal ions in the electrolyte are always solvated by solvent molecules and anions, which is highly related with the electrochemical performance [103, 104]. During the solvation process, the anions and solvent molecules compete for coordination with Li^+^ ions through ion–ion and ion–dipole interactions, respectively. Then, it could form solvation complexes with specific components and structures, which are called solvation structures [31, 105]. When weaken the solvation structures, the transport behavior of charge carriers could be suppressed, leading to the insufficient electrochemical performance. Wang et al. regulated the solvation structure of Li^+^ by modifying the electrolyte components, optimization of the SEI structure to achieve dendrite-free lithium deposition [106]. It was observed that the ion–dipole interactions occurring between the electron-deficient B atoms present in lithium difluoroborate (LiDFOB) and the O atoms within the dimethoxyethane (DME) solvent molecule played a significant role. These interactions served to weaken the binding forces between the DME molecule and Li^+^. As a consequence, the dissolution process of Li^+^ was accelerated. Building upon this discovery, it was further determined that the ion–dipole interaction had the power to drive a substantial number of anions into the inner solvated sheath layer surrounding Li^+^. This, in turn, actively promoted the formation of SEI that was rich in inorganic components, which was crucial for realizing the goal of dendrite-free lithium deposition.

**Fig. 7:**
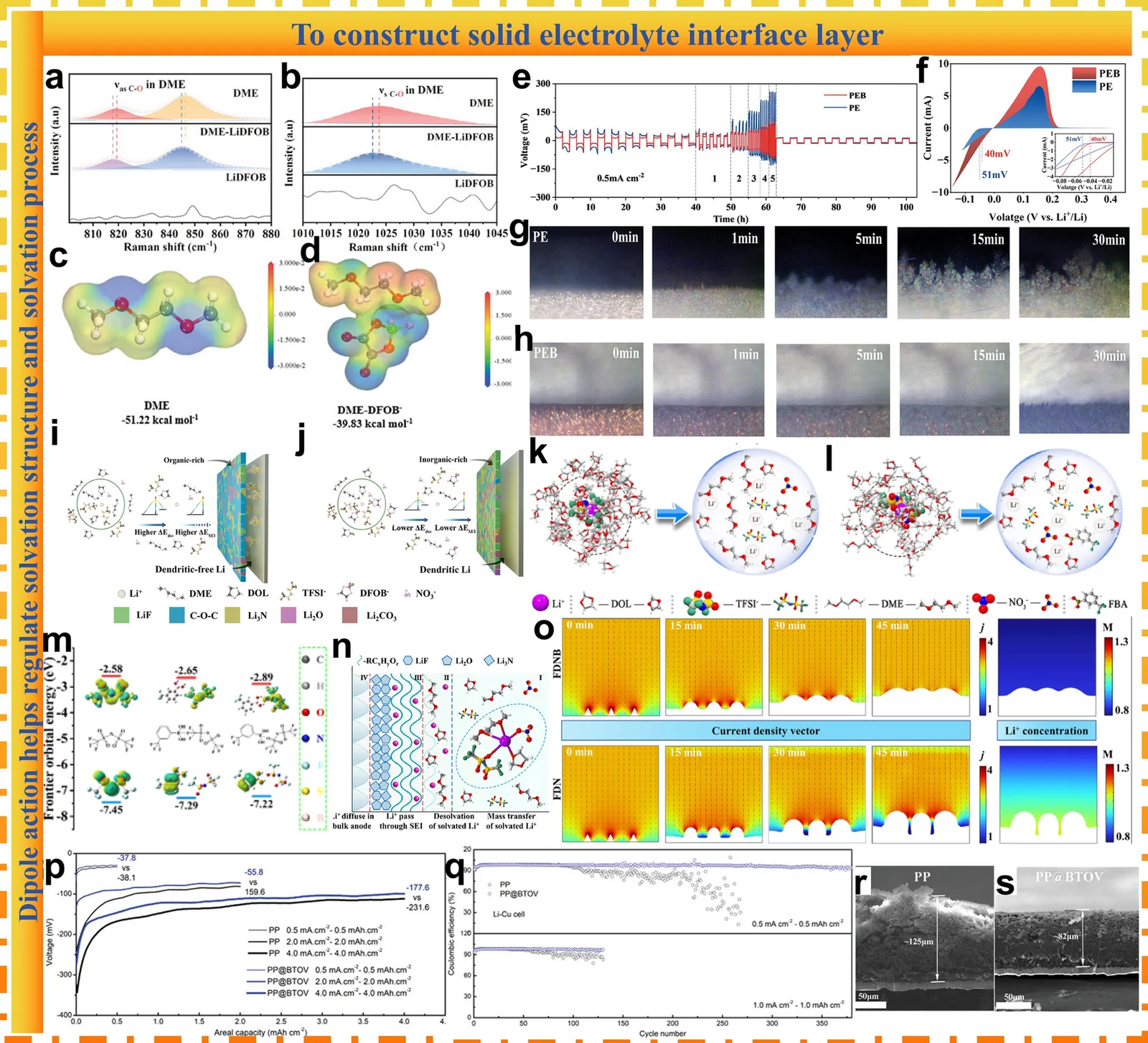
**a** C–O asymmetric stretching mode and **b** C–O symmetric stretching mode obtained from Raman spectra of pure DME and DME-LiDFOB complex. EPM of **c** DME and **d** DME-DFOB^−^ complex. **e** Rate performance of Li||Li cells with PE and PEB electrolytes. **f** CV curves of Li||Cu cells with PEB and PE electrolytes. In situ optical microscopy observations of Li deposition process in **g** PE and **h** PEB electrolytes. Schematic representation of the solvated structure and the electrolytes–anode interface in **i** PE and **j** PEB electrolytes. Reproduced with permission from Ref. [106]. Copyright 2024 Wiley–VCH GmbH. The schematic diagrams of Li^+^ ion local solvation environment in **k** FDN and **l** FDNB electrolytes. **m** Molecular orbital energies of TFSI^−^ anion before and after interacting with FBA molecule via B-F and B-O; blue line: HOMO energy level, red line: LUMO energy level. **n** Li^+^ ion transfer behavior in electrolyte during charge process. **o** Current density vector profiles and Li^+^ ion concentration distribution in FDN and FDNB electrolytes, j: mA cm^−2^, M: moL L^−1^. Reproduced with permission from Ref. [112]. Copyright 2023 Elsevier B.V. **p** Voltage versus capacity plots of Li-Cu cells. **q** CEs of Li-Cu cells under a current density of 0.5 mA cm^−2^ with a capacity of 0.5 mAh cm^−2^ (upper panel) and under a current density of 1.0 mA cm^−2^ with a capacity of 1.0 mAh cm^−2^ (lower panel). **r** Pure PP separator and **s** PP@BTOV separator after cycling. Reproduced with permission from Ref. [113]. Copyright 2024 Wiley–VCH GmbH

As depicted in Fig. 7a, b, the typical bands positioned at 819.3 and 846.2 cm^−1^ were associated with the asymmetric stretching mode of C–O asymmetric, while the typical band at 1023.6 cm^−1^ corresponds to the symmetric stretching mode of C–O asymmetric in pure dimethoxyethane (DME) [107–110]. After the introduction of lithium difluoroborate (LiDFOB), it was noticed that these bands underwent a slight red-shift. This phenomenon could be ascribed to the ion–dipole interaction taking place between the electron-deficient B atom within the DFOB- anion (B_DFOB⁻_) and the O atom of the DME molecule (ODME) [111].

The minimum electrostatic potential (EPM) of the solvent could be used as a descriptor to qualitatively determine the solvation strength. According to the calculation results, the EPMs of pure DME and the DME-DFOB- complex are −51.22 and −39.83 kcal mol^−1^, respectively (Fig. 7c, d). Compared to pure DME, the EPM of the DME-DFOB^−^ complex dropped significantly, further demonstrating that the interaction between BDFOB- and ODME lessens that between the DME molecule and Li^+^. Moreover, the desolvation energy of Li^+^ coordinated with the DME molecule and the DME-DFOB^−^ complex was calculated. Results revealed that the desolvation energy of Li^+^ decreased from 0.482 eV for DME to 0.188 eV for DME-DFOB^−^, aligning with the trend calculated by EPM. The outcomes of EPM and Li^+^ desolvation energy suggested that the ion–dipole interaction between ODME and BDFOB^−^ weakens the DME-Li^+^ interaction, reduces Li^+^ desolvation energy, and expedites the Li^+^ desolvation process.

The electrochemical performances of different electrolytes for LMBs were investigated. The performance rate is shown in Fig. 7e. The results indicated that at high current densities, the cells displayed significant voltage fluctuations in the PE electrolyte. In contrast, at all current densities, the cells in the PEB electrolyte possessed a relatively flat voltage plateau. The enhancement of the high-rate nucleation and deposition behavior of Li^+^ within the PEB electrolyte could be ascribed to the formation of a sturdy SEI with high ionic conductivity, which was brought by the addition of LiDFOB. The results of the cyclic voltammetry (CV) curves of the Li||Cu cells also reflected this tendency. As illustrated in Fig. 7f, the Li^+^ plating over-potential detected in the PEB electrolyte was 40 mV, which was considerably lower than that in the PE electrolyte (51 mV). This further demonstrated that the deposition kinetics of Li^+^ in the PEB electrolyte had been optimized.

The changes in the electrode morphology during the Li^+^ deposition process in the two electrolytes were observed by in situ optical microscopy, convincingly demonstrating the key role of the modified solvation structure in promoting the formation of a stable SEI. As shown in Fig. 7g, dendrites began to appear after 5 min of deposition in the PE electrolyte and grew rapidly during the subsequent deposition process, forming a dendrite cluster after 30 min of deposition. In the PEB electrolyte, the deposition maintained a smooth morphology, and no obvious dendrite formation was observed during the entire deposition process (Fig. 7h). These differences could be attributed to LiDFOB, weakening the interaction between dimethyl ether and Li^+^, reducing the solvation energy barrier of Li^+^, thus regulating the solvation sheath to form an inorganic-rich SEI and inhibiting the formation of Li dendrites (Fig. 7i, j).

Attributed to the fact that Li^+^ tends to bind to solvent molecules and anions in the electrolyte, solvated components close to the Helmholtz plane were more susceptible to gaining electrons from the Li electrode and were preferentially reduced under the influence of the electric field. Therefore, the solvated structure provided a molecular basis for regulating the structure and composition of the interfacial phase at the electrode–electrolyte interface and thus constructing a stable SEI layer. Liu et al. designed an anion receptor, which played a significant role in enhancing the contribution of nitrate anions to the formation of the SEI [112]. Figure 7k, l presents the typical local solvation environments of lithium ions in the FDN and FDNB electrolytes. In the FDNB electrolyte, Li^+^ was prone to form contact ion pair (CIP) and aggregate (AGGs) configurations with anions, namely, TFSI^−^ and NO_3_^−^. By contrast, in the FDN electrolyte, Li^+^ tends to coordinated with solvent molecules such as dimethoxyethane (DME) and 1,3-dioxolane (DOL). Figure 7m illustrates the frontier molecular orbitals of the TFSI^−^ anion under different coordination structures within the electrolyte. Thanks to interaction between the FBA molecule and the TFSI^−^ anion via the formation of B–O and B–F bonds, the reduction stability of the TFSI^−^ anion was substantially decreased. This decrease was beneficial for the generation of the SEI that features high mechanical strength, high interfacial energy, and excellent electrical insulation properties, which were of great importance for ensuring excellent performance and stable operation of the battery.

In principle, the transfer behavior of Li^+^ in the electrolyte could be categorized into the following four stages during the charging process. (I) Movement of solvated Li^+^ in the bulk electrolyte by mass transfer; (II) desolvation process of solvated Li^+^ on the SEI surface; (III) desolvated Li^+^ passing through the SEI; and (IV) desolvated Li^+^ diffusing into the bulk electrode and obtaining electrons to be reduced (Fig. 7n). Throughout these processes, two pivotal barriers exerted a substantial impact on the transfer kinetics of lithium ions within the electrolyte and their subsequent deposition behavior were the desolvation barrier encountered by solvated Li^+^ and the diffusion barrier faced by desolvated Li^+^ as they traverse the SEI.

The presence of fast ionic transfer kinetics and a uniform flux of Li ions play a crucial role in facilitating a uniform distribution of both the concentration gradient and the electric field at the interface during the Li ion plating process. In this research, finite-element analysis (FEA) was employed to simulate the dynamic changes in the Li^+^ concentration gradient as well as the electric field distribution throughout the Li deposition process (Fig. 7o). In the FDN electrolyte, as the deposition time increases, a high current density can be observed at the tip of the Li protrusion. This current density was much higher than that in the other areas. Such a non-uniform distribution of the current density gives rise to the significant growth of Li dendrites. On the contrary, the FDNB electrolyte demonstrated a relatively uniform distribution of surface current. This uniformity effectively suppressed the tip effect during the Li deposition process, thereby reducing the likelihood of Li dendrite formation.

In addition, according to the evolution law of the concentration field, the diffusion of ions in different electrolytes was studied. For the FDN electrolyte, the distribution of Li^+^ on the electrode surface provided a typical concentration polarization region. Moreover, the concentration distribution of Li^+^ on the electrode surface in the FDN electrolyte was non-uniform, concentrating at the protruding tips. The non-uniform concentration distribution of Li^+^ on the Li electrode could lead to the selective deposition of Li^+^ in the vertical direction, called dendritic deposition. In sharp contrast, in the FDNB electrolyte, the Li^+^ on the electrode surface was uniformly distributed, so the concentration polarization was significantly suppressed. The concentration distribution of Li^+^ on the electrode surface in the FDNB electrolyte was more uniform than that in the FDN electrolyte. The uniform concentration distribution could be attributed to the fast ion migration ability and uniform Li^+^ flux in the FDNB electrolyte. In the FDNB electrolyte, the uniform concentration distribution of Li^+^ on the electrode surface allowed uniform Li deposition, which was crucial for achieving an efficient and safe lithium–metal electrode.

Therefore, Xu et al. focused on a separator coated with dipole acting molecules [113], which also effectively solved the problem of lithium dendrite deposition. As depicted in Fig. 7p, for the Li–Cu cells with the PP@BTOV separator at current densities of 0.5, 2.0, and 4.0 mA cm^−2^, the nucleation overpotentials were 37.8, 55.8, and 177.6 mV, respectively, all of which were lower than those of the pure PP separator (38.1, 159.6, and 231.6 mV), indicating a reduced Li nucleation barrier upon introducing the BTOV layer. Moreover, the cells using the PP@BTOV separator exhibited lower plating potentials during continuous plating, suggesting that the SEI formed has lower impedance. In addition, the average Coulombic efficiencies (CE) of the cells using the BTOV separator were 97.0% (380 cycles) and 97.7% (130 cycles) at 0.5 mA, −0.5 mA cm^−2^/0.5 mAh^−2^, and 1.0 mA cm^−2^/1.0 mAh^−2^, respectively, significantly higher than those of the cells with the pure PP separator (Fig. 7q). After 100 cycles at 1.0 mA cm^−2^/1.0 mAh^−2^, the deposition thickness can reach 125 μm. For the cells using the PP@BTOV separator, the deposited Li had a smoother and denser morphology, and its thickness was much thinner compared to the cells using the PP separator (Fig. 7r, s). This was because the BTOV layer with an ordered dipole arrangement can regulate the Li^+^ flux near the anode, thus facilitating uniform Li deposition and stripping, and suppressing the growth of dendrites and the formation of "dead" lithium.

Metal-ion batteries are unstable at high temperatures for a variety of reasons. In addition to the chemical stability of the material itself, the most significant element is the solvated structure of the electrolyte [114–119]. It greatly defines the SEI/CEI characteristics, charge transfer behavior, and desolvation energy barriers, which are essential to achieve high cycling stability of LMBs [120–124]. Taking lithium-ion batteries as an example, usually, lithium salts dissolve in solvents, driven by ionic dipole interactions between lithium ions and solvent molecules (e.g., carbonates, ethers, etc.). These noncovalent interactions give rise to different solvated structures. However, at high temperatures, the ion–dipole interactions are significantly weakened [125–127]. Consequently, the solvation structure is subsequently disrupted and cannot form an efficient and stable SEI layer, thereby resulting in excessive side reactions and battery failure [128, 129]. Therefore, designing an electrolyte that can maintain a good solvation structure and form a stable SEI layer at high temperatures is of great importance for the development of high-energy batteries. The molecular ion–dipole interaction can effectively address such issues. Chen et al. designed a heat stable electrolyte with a steady solvated structure based on multiple ionic dipole interactions [130]. Strong ligands in the solvated structure of the electrolyte determined the lithium deposition behavior at high temperatures and the evolution of the solid–electrolyte interfacial phase, which was key to achieving higher lithium Coulombic efficiency and avoiding lithium dendrite growth.

**Fig. 8:**
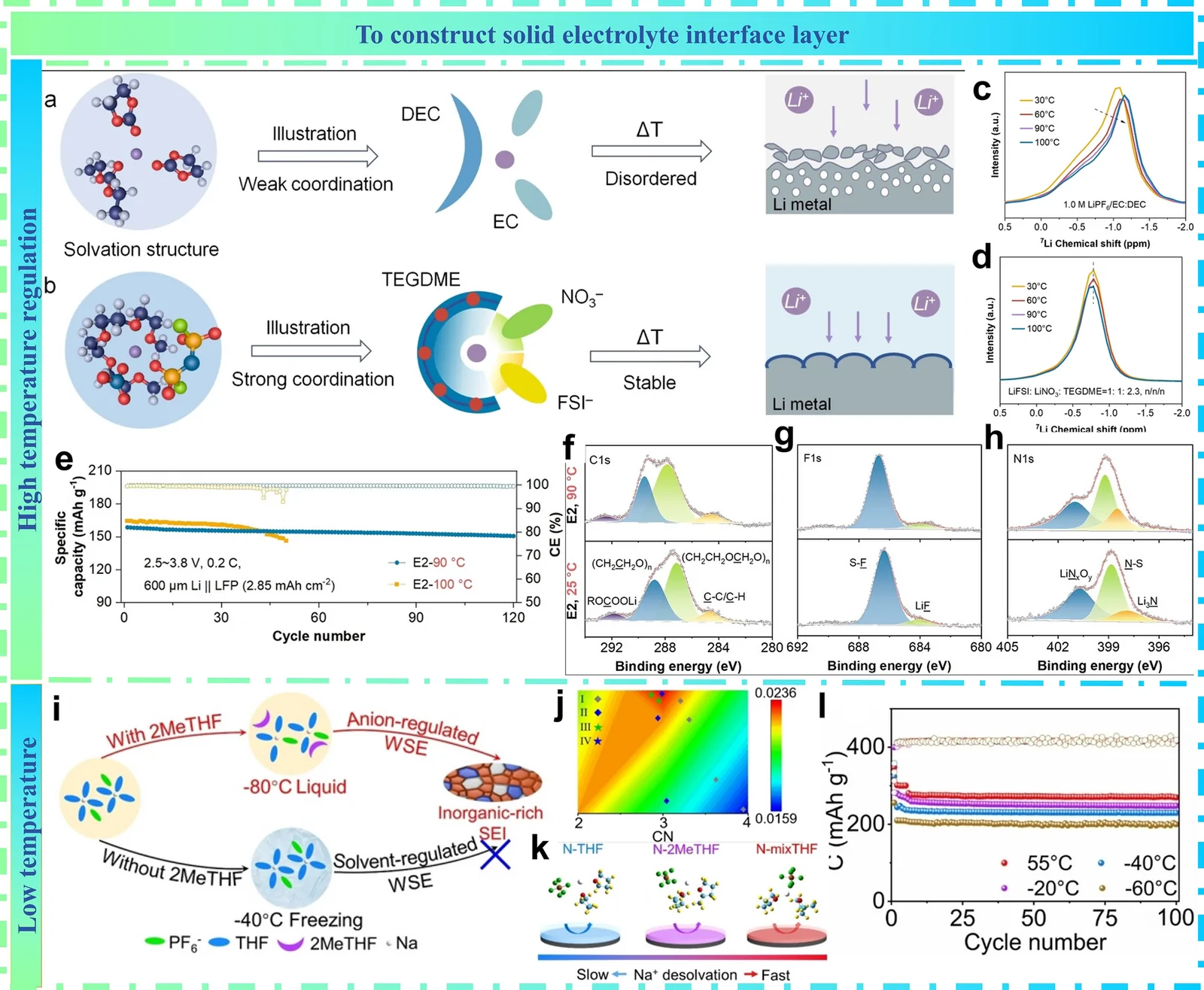
Schematic diagrams of lithium deposition at elevated temperatures with different solvation structures in **a** conventional ester electrolyte (1.0 M LiPF_6_/EC: DEC) and **b** LiFSI/LiNO_3_/TEGDME electrolyte. **c** ^7^Li NMR of 1.0 M LiPF_6_/EC: DEC electrolyte and **d** LiFSI/LiNO_3_/TEGDME electrolyte at 30–100 °C. **e** Cycling stability of Li‖LFP cells-based E2 electrolyte at 90 °C and 100 °C. XPS analysis of the Li anodes from Li|E2|LFP cells after 10 cycles at 0.1 C in 25 °C and 90 ℃. **f–h** C 1*s*, F 1*s,* and N 1*s* XPS spectra of the two cycled Li anodes. Reproduced with permission from Ref. [130]. Copyright 2022 Wiley–VCH GmbH. **i** Mechanism of Na^+^-dipole regulation on formation of inorganic-rich SEI. **j** Calculated electron density of BCP for Na^+^-O bond in three electrolytes. **k** Schematics of different Na^+^ desolvation at HC anode surface. **l** Long cycling performance in N-mixTHF at different temperatures (100 mAg^−1^ beyond − 20 °C, 50 mAg^−1^ at − 40 °C, and − 60 °C**)**. Reproduced with permission from Ref. [59]. Copyright 2024 Wiley–VCH GmbH

Under high-temperature conditions, since the desolvation energy barrier of solvation was prone to disordering, many lithium dendrites and dead lithium were generated, and side reactions were increased (Fig. 8a). In contrast, the solvation structure with strong ion–dipole interactions could remain stable under high-temperature conditions (Fig. 8b). The relatively higher desolvation energy barrier for lithium ions promoted the more uniform deposition of lithium ions, thereby enhancing the cycling stability of the battery.

The solvation structure which relies on multi-ion–dipole interactions possessed outstanding high-temperature stability. The solvation structures at different temperatures were investigated using nuclear magnetic resonance (NMR) measurement methods. The change in the electronic environment of the ^7^Li nucleus indicates the change in the lithium-ion solvation structure [131, 132]. As shown in Fig. 8c, under high temperature, the ^7^Li chemical shift in the LiPF_6_/EC/DEC system shifts toward a higher field (more negative field), corresponding to the gradual dissociation of the solvation structure. In sharp contrast, even when the temperature rises to 100 °C, the ^7^Li chemical shift of the LiFSI/LiNO_3_/TEGDME electrolyte does not change (Fig. 8d). These results verified the high thermal stability of multiple ion–dipole interactions during the solvation process. Even at 10 °C, the battery can undergo 50 cycles at a rate of 0.2 C, achieving a capacity retention rate of 89% (Fig. 8e). Compared with the situation at 100 °C, the battery’s polarization increases slightly only at 90 °C, which was attributed to the elevated loss of active lithium. To further verify the interface stability between the E2 electrolyte and the Li anode, we contrasted the XPS spectra of a 50 μm Li anode at 25 and 90 °C. The atomic ratios of oxygen (O), fluorine (F), sulfur (S), and nitrogen (N) in the SEI formed at 90 °C exhibited merely slight variations compared to those in the SEI formed at 25 °C. The Li content drops from 46.2% at 25 °C to 42.2% at 90 °C, while the carbon (C) content rises from 13.9% at 25 °C to 16.1% at 90 °C. The XPS spectra revealed that only (CH_2_CH_2_O) n and lithium nitride show a slight increase, with no significant alterations in the rest (Fig. 8f–h). This also suggested that the E2 electrolyte maintains a stable solvation structure even under high-temperature conditions. This was due to the robust ion–dipole interactions within the electrolyte, thus ensuring good interface stability. In addition, in low-temperature environments, the solvation structure could also be stabilized and intervened through dipole interactions, enabling the battery to maintain good electrochemical performance even at low temperatures. Fang et al. designed a weakly solvating electrolyte (WSE) with reduced ion–dipole interactions for stable sodium storage in a hard carbon (HC) anode at ultra-low temperatures [59]. It maintained an anion-enhanced solvation structure from room temperature to low temperatures to promote the formation of an inorganic-rich SEI (Fig. 8i).

They adopted the topological electronic density analysis method to compare the Na^+^–dipole interactions in different electrolytes. Bond critical points (BCPs) were used to identify the electron density distribution and bonding characteristics in the solvation structure [133]. The BCPs of the Na^+^-solvent bonds and their corresponding electron densities (*ρ*_e_) are shown in Fig. 8j. The *ρ*_e_ values of Na^+^-OTHF and Na^+^-O_2MeTHF_ in N-mixTHF are 0.05758 and 0.01866, respectively, which were lower than those of Na^+^-OTHF in N-THF (0.07235) and Na^+^-O_2MeTHF_ in N-2MeTHF (0.04561). The lower *ρ*_e_ value of Na^+^–O solvent indicated a weakened ion–dipole interaction between Na^+^ and the solvent. This leads to more anions participating in the solvation sheath and improves the Na^+^ desolvation kinetics, as shown in Fig. 8k. Figure 8l presents the electrochemical behavior of the hard carbon (HC) anode in the N-mixTHF electrolyte from 80 to 55 °C. At 60 °C, the hard carbon anode exhibited a high specific capacity of 205.4 mAh g^−1^, retaining 76.1% of the capacity obtained at 25 °C. Even in the N-mixTHF electrolyte at 80 °C, a reversible specific capacity of 58.3 mAh g^−1^ could be achieved. In addition, the hard carbon anode based on N-mixTHF had a capacity retention rate higher than 95% after 100 cycles in the temperature range from 80 to 55 °C, and the average Coulomb efficiency reached 99.9%, indicating that N-mixTHF had excellent adaptability in both high- and low-temperature environments. This excellent performance was attributed to the weakened ion–dipole interaction and the enhanced solvation structure of anions, which promoted the Na^+^ desolvation process and the formation of the inorganic-rich SEI on the anode, thereby bringing better performance than previously reported low-temperature electrolytes. This study emphasized the importance of the solvation structure influenced by ion–dipole interaction at elevated temperatures.

### Heightening the Electrical Double Layer

Since the dipole interaction could contribute to the construction of a stable electrical double layer (EDL), Huang et al. designed a polar propylene carbonate (PC) with a strong dipole interaction [46]. The polar PC was paired with tetrafluoroborate anions, establishing a strong ion–dipole interaction. The strong ion–dipole interaction could not only modify the solvation structure of zinc ions, but also facilitate the formation of a dynamic electrical double layer on the surface of the zinc electrode, suppressing the formation of the ZnF_2_ interface and carbonates, and thus promoting the uniform deposition of zinc ions.

**Fig. 9:**
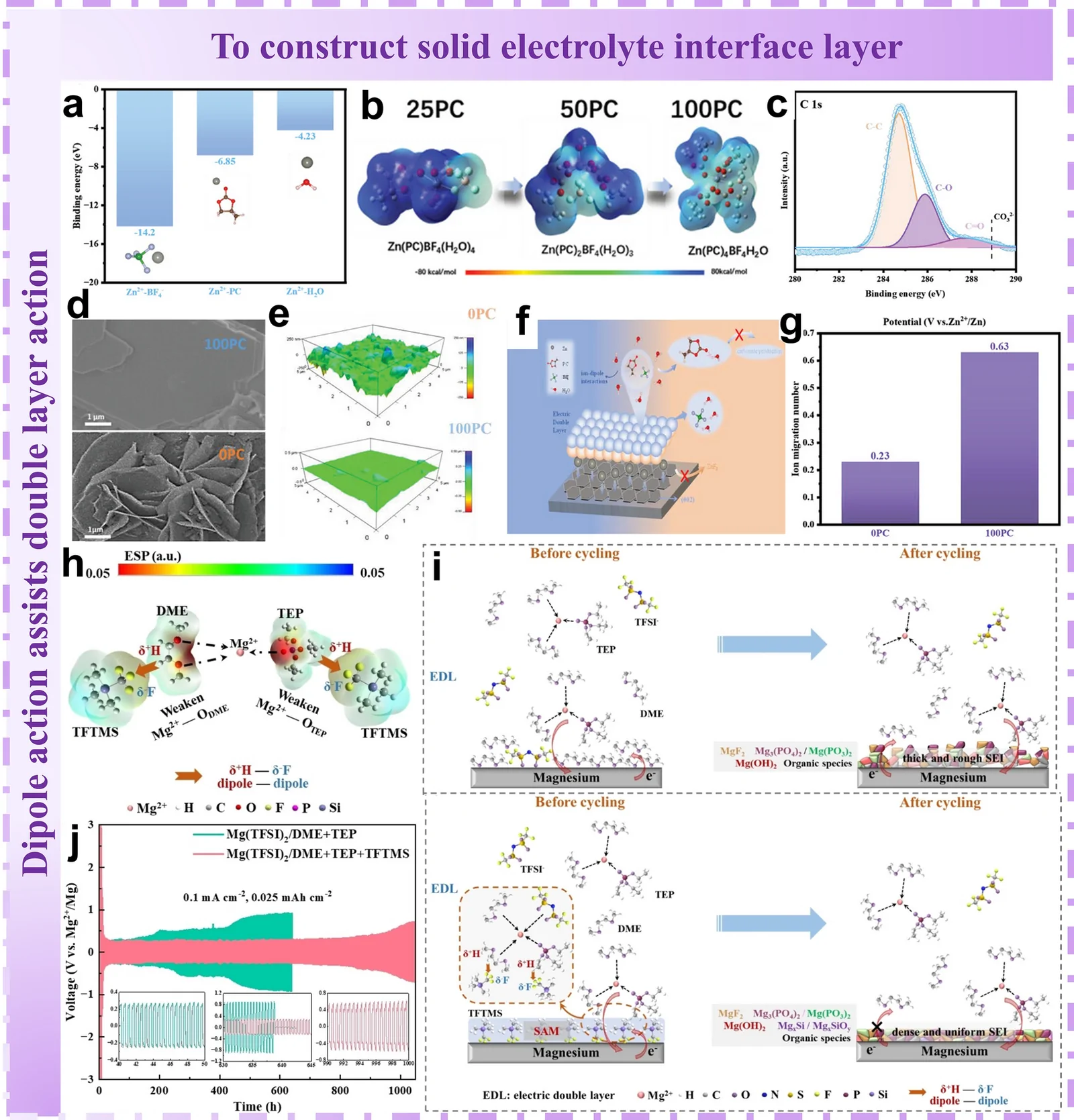
**a** Binding energy between different molecules and ions. **b** Electrostatic potential of the solvation structures in different systems. **c** C 1s spectrum of Zn foil. **d** SEM images of the zinc foil after 20 h of cycling in 0 and 100 PC electrolytes. **e** Three-dimensional height images of Zn substrate after deposition in different electrolytes. **f** Mechanism diagram of the effect of strong ion–dipole interaction. **g** Migration number of zinc ions in different electrolytes. Reproduced with permission from Ref. [46]. Copyright 2024 Wiley–VCH GmbH. **h** The effect of TFTMS on dipole–dipole interactions among surrounding solvent molecules (DME, TEP) and their coordination with Mg^2+^. **i** Mechanism diagram of TFTMS additives to construct SAM and modulate SEI nanostructure. **j** Voltage profiles at 0.1 mA cm^−2^ with 0.025 mAh cm^−2^. Reproduced with permission from Ref. [135]. Copyright 2024 Wiley–VCH GmbH

Strict DFT calculations demonstrated that the binding energies of Zn^2+^ with BF_4_- and PC molecules were significantly higher than those of Zn^2+^ with water molecules, as shown in Fig. 9a. Among which, the solvation and desolvation processes of zinc ions in water-based electrolytes are crucial to their deposition characteristics, in which water plays an important role. This finding indicated that in the solvation structure of zinc ions, they are more likely to bind with PC molecules and BF_4_^−^. In addition, the DFT result showed that the binding energy between BF_4_- and PC molecules was higher than that of PC-H_2_O and BF_4_-H_2_O interactions. This implies that the ion–dipole interaction between BF_4_^−^ and PC was more stable, thereby preventing direct contact among PC molecules, BF_4_^−^, and water molecules, and suppressing the formation of undesirable by-products. In the solvation structure, it decreases with the increase in PC content, because the electrostatic potential of the zinc ion solvation structure was affected by the interaction between anions and dipole molecules. Specifically, BF_4_^−^ attracts the positive charge center within PC molecules, thereby weakening the electric field strength of PC (Fig. 9b). Meanwhile, the weak hydrogen bonds among water molecules gradually decrease, and the interaction of BF_4_^−^H_2_O and PCBF_4_- gradually played a dominant role. This observation indicates that due to the presence of BF_4_^−^, the ion–dipole interaction suppressed the activity of water molecules in the electrolyte. Furthermore, the interaction between BF_4_- and PC established a stronger ion–dipole interaction.

The C–C, C–O, and C–O signal peaks in the C 1*s* spectrum could be attributed to PC molecules adsorbed on the surface of the Zn electrode, while the signal peak representing CO_3_^2−^ does not appear in the pattern (Fig. 9c). There were also no C–O and Zn–O signals in the spectrum. The double-layer capacitance curves obtained at different scanning rates showed that the capacitance of the 100 PC electrolyte was lower than that of the 0 PC electrolyte. This phenomenon could be attributed to the replacement of water molecules within the electrical double layer by PC molecules due to the strong ion–dipole interaction of BF_4_ [134]. This layered structure could not only avoid the contact between water molecules and PC to generate carbonates, but also prevent the reaction between BF_4_- and water on the surface of the zinc electrode to form by-products, such as ZnF_2_. In summary, a dynamic electrical double-layer structure was formed on the surface of the PC molecules and the BF_4_- on the zinc electrode. This structure not only promoted the interaction between the zinc electrode and water molecules, but also facilitated the directional deposition of zinc ions.

From the scanning electron microscopy (SEM) images (Fig. 9d), zinc was uniformly distributed on the surface of the zinc foil and stacked along the (002) crystal plane. Through atomic force microscopy, the deposition states of zinc in the two electrolytes could also be observed. The roughness of the zinc foil in the 100 PC electrolyte was only 12 nm. The surface of the zinc foil was more uniform and flatter because zinc ions were deposited along the (002) surface. However, the roughness of the zinc foil in the 0 PC electrolyte reaches 26 nm. Obviously, the zinc surface in the 0 PC electrolyte was rough with many dendrites. These numerous dendrites could easily penetrate the separator layer, leading to a short circuit, which significantly reduced the cycle life (Fig. 9e). The results clearly showed that BF_4_- and PC molecules form a stable double-layer structure on the surface of the zinc electrode. This structure not only enhanced the solvation structure of zinc ions, but also prevented the formation of carbonates and zinc fluoride. Therefore, this promotes the uniform deposition of zinc ions along the (002) surface, thereby improving the stability of zinc deposition (Fig. 9f). The mobility of the electrolyte in the 100 PC electrolyte was significantly higher than that of the 0 PC solution (0.23) (Fig. 9g). This could be attributed to the improvement of the solvation structure of zinc ions and the formation of a stable electrical double layer, which helped in the construction of a stable SEI layer, thereby promoting the formation of favorable transport pathways. The reduction in free water molecules in the electrolyte, combined with the coordination effect of PC molecules, BF_4_-, and Zn^2+^, contributed to the enhancement of the zinc ion transfer kinetics. Thus, the dipole interaction helped the electrical double layer to induce the formation of the SEI layer, thereby reducing the occurrence of side reactions, making the deposition of ions at the negative electrode interface more uniform, and suppressing the generation of dendrites that could cause a decline in battery performance.

Similarly, Fan et al. proposed a self-assembled monolayer (SAN) regulation strategy [135]. They introduced a small number of functional adsorbents into the electrolyte to form a self-assembled monolayer on the surface of magnesium metal. By utilizing the dipole–dipole interaction between the adsorbents and solvent molecules to regulate the electrical double layer, the construction of a stable SEI was achieved. From the perspective of electrostatic potential (Fig. 9h), the DME (dimethoxyethane) and TEP (triethyl phosphate) molecules were more electron-deficient on H (in CH_2_ or CH_3_ groups), while the TFTMS (trifluorotrimethylsilane) molecule was more electron rich on F (in CF_3_ groups). A dipole–dipole interaction was formed between the positively charged hydrogen (δ^+^H) and the electronegative fluorine (δ^−^F) of DME/TEP and TFTMS. Therefore, the downfield shifts of CH_2_ and CH_3_ in DME molecules were attributed to the dipole–dipole interaction between DME and TFTMS. The addition of TFTMS weakens the synergy of DME. Meanwhile, the downfield shifts of CH_2_ and CH_3_ in TEP molecules could be attributed to the decrease in Mg^2+^-OTEP and the weakened dipole–dipole interaction between TEP and TFTMS [136]. Based on the above experiments and calculations, the remarkable effect of the strategy was mainly attributed to the formed self-assembled monolayer (SAM). It modulates the Mg^2+^ coordination environment in the electrical double layer (EDL) and optimizes the structure of the electrical double layer, thus generating a stable SEI on the Mg metal anode (Fig. 9).

Regulating the EDL on the anode surface by forming a SAM on the Mg metal was beneficial for the adsorption of the strong electron-withdrawing group CF_3_ onto Mg. Specifically, through the inductive effect between the electronegative fluorine (^δ−^F) and the positively charged hydrogen (^δ+^H), the dipole–dipole interaction between TFTMS and DME/TEP was generated. It adjusts the Mg^2+^ solvation sheath in the EDL and influences the structure of the electrical double layer within the SEI layer. Finally, due to the optimized solvation configuration, a stable and dense fluorine- and phosphate-containing SEI was assembled on the Mg metal electrode. Precisely because of this, the environment of the electrical double layer was influenced by the dipole interaction, which consequently induced the formation of a stable SEI. As a result, the cycling life of the symmetric cell was significantly improved. After modification, the cycling life exceeds 1000 h, while it was only approximately 500 h before the modification (Fig. 9). Importantly, the optimized electrolyte not only ensured stable and long-life, low polarization cycling of symmetric cells, but also exhibited good compatibility with different cathode materials. More importantly, this strategy demonstrated its universality and was applicable to the practical applications of other electrolyte systems.

### Optimizing the Structure of SEI

**Fig. 10:**
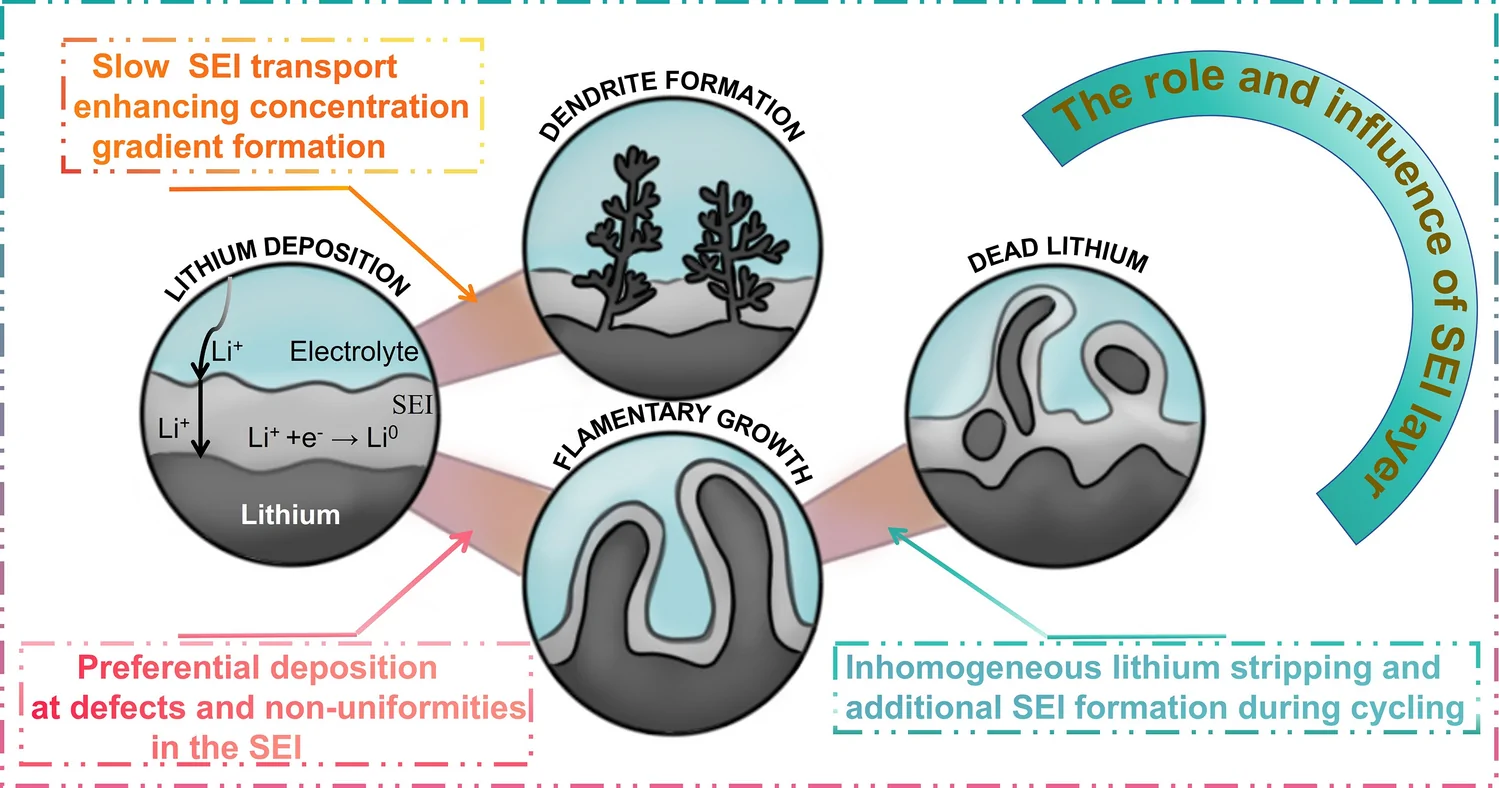
The role and influence of SEI layer

The SEI film possessed excellent ionic conductivity, allowing lithium ions to shuttle freely within the battery [137–139]. It was ingeniously positioned between the electrodes and the electrolyte of the battery, serving to prevent excessive progress of internal chemical reactions in the battery and effectively suppressing the formation of metal-ion dendrites (Fig. 10). Therefore, constructing the SEI layer was an effective approach to solving the dendrite problem. The electrical double layer on the electrode surface and the solvation behavior determined the composition and structure of the SEI layer. Moreover, the dipole interaction was conducive to regulating the electrical double-layer environment and interfering with the solvation behavior. Therefore, constructing an effective SEI layer through dipole interaction was beneficial for resolving the dendrite problem on the negative electrode surface, thereby enhancing the electrochemical performance and stability of the battery [140–142].

In addition to assisting ion transport, optimizing the electric double-layer and solvation structure, molecular dipole could also be designed and optimized to form a stable SEI layer to improve the electrochemical performance of the battery. Zhang et al. designed a composite solid electrolyte (PHMP) by leveraging the dipole interaction to construct an SEI layer that facilitated ionic transport and has good stability [64]. This electrolyte effectively inhibited metal-ion dendrites.

**Fig. 11:**
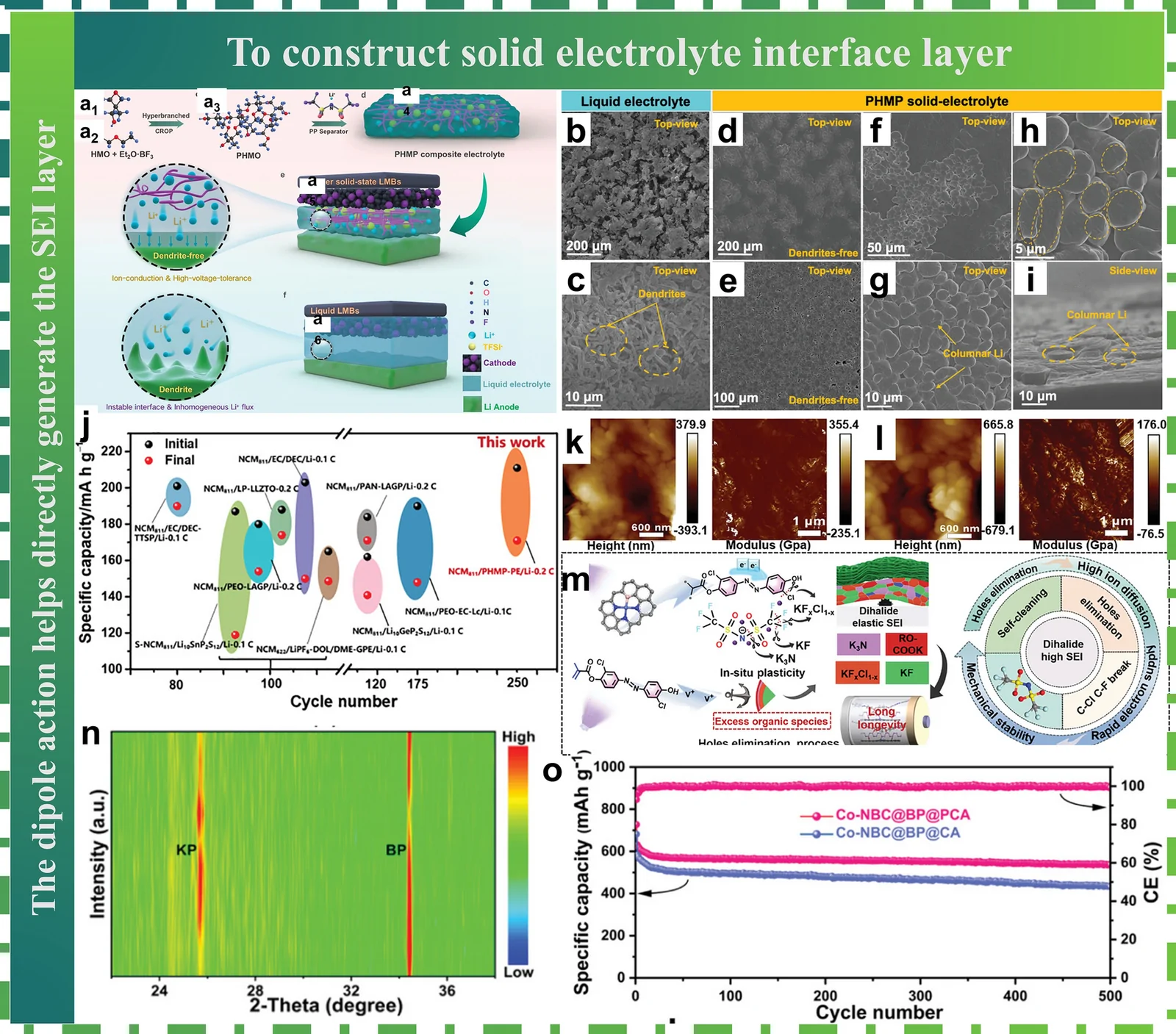
**a** Synthetic scheme of PHMP composite solid electrolyte through (**a1–a3**) CROP, (**a4**) separator compositing, and (**a5**, **a6**) the uniform Li^+^ ion diffusion and deposition enabled by PHMP. Formation of LiF-rich SEI and columnar Li deposition by PHMP: **b**, **c** SEM images of Li deposition in liquid electrolytes; **d**–**i** top-view and side-view SEM observation of columnar Li deposition in PHMP-modified cells. **j** Comparison of the electrochemical performance of NCM811| Li cells in other liquid or SSE reported in the literature. Reproduced with permission from Ref. [64]. Copyright 2021 Wiley–VCH GmbH. **k** Co-NBC@BP@PCA when immerged in electrolytes (PIBs) under UV light illumination, and **l** BP when discharged to 0.7 V. **m** Schematic illustration of the SEI evolution for Co-NBC@BP@PCA. **n** Corresponding in situ XRD profiles for Co-NBC@BP@PCA. **o** Cycling performance at a current density of 2 A g^−1^. Reproduced with permission from Ref. [146]. Copyright 2024 Wiley–VCH GmbH

Through further synthesis with a polypropylene separator, a self-supporting polymer composite electrolyte (PHMP) was obtained, which has the characteristics of flexibility, rigidity, and a low glass transition temperature (*T*_g_ = −36.4 °C). Molecular design (polymer hyperbranching) verified its effective polymer amorphization. Moreover, first-principles simulations showed that the hyperbranched PHMP introduced an “ion–dipole” configuration as a lithium-ion coordination solvation cage, enhancing salt dissociation and ion conduction, while suppressing the decomposition process triggered by protons under high voltage (Fig. 11a). As shown in Fig. 11b, liquid electrolyte cells generated a rough and loose lithium surface due to excessive side reactions, dead lithium formation, and volume expansion. Moreover, many filamentous dendrites aggravated the interfacial instability (Fig. 11c) [143, 144]. In contrast, the surface induced by PHMP was smooth and dense without protrusions (Fig. 11d–f). The top-view images in Fig. 11g, h displayed uniform columnar lithium deposition, indicating different SEI compositions. The side-view image in Fig. 11i confirmed the growth of columnar lithium, and its large diameter-to-length ratio resulted in a thin and compact interface. Compared with lithium whiskers, massive lithium deposition could suppress continuous side reactions caused by free solvent decomposition. The morphology of lithium deposition depended mainly on the concentration and diffusion rate of lithium ions at the interface, following the diffusion reaction competition mechanism [145]. When the interfacial reaction was controlled by the diffusion step (i.e., slow ion diffusion), lithium was deposited as dendrites. When the reaction control dominates (i.e., fast ion diffusion), stable columnar or spherical depositions form. Thus, an ideal SEI component with high surface energy and a low lithium-ion diffusion barrier was crucial for the rapid diffusion of lithium ions. According to the corresponding results in the literature, the electrochemical performance of PHMP-modified cells was far superior to that of some reported NCM811|Li cells (Fig. 11j), highlighting the progress of PHMP in supporting high-voltage LMBs. Therefore, the PHMP designed by Zhang et al. provided an idea for directly constructing the SEI layer of high-energy batteries.

Utilizing the dipole interaction of molecular ions to directly design the SEI layer, with the enhancement of the interaction between functional groups and metal salts, was also an effective approach in the realm of battery research. He et al. proposed a dipole–dipole interaction between polar groups (–COO–, –N=N–, and –OH–) and metal salts [146]. The elastic organic outer layer significantly enhanced the desolvation kinetics, formed intermolecular hydrogen bonds between PCA (propylene carbonate) and DME (dimethoxyethane), and introduced high steric hindrance due to the side chain segments. Meanwhile, these polar groups provided a fast side chain carrier transport channel. Compared with traditional methods, by utilizing the dipole ultraviolet light-induced in situ strategy, a high-performance polymeric SEI with high uniformity, elasticity, and flame retardancy was successfully constructed through a simple and effective approach.

The SEI layer of Co-NBC@BP@PCA had a relatively smooth Young's modulus of 355.4 GPa, while the rough surface of BP (Fig. 11k) has a Young's modulus of 176 GPa according to atomic force microscopy (AFM), which further supported the uniformity and excellent mechanical stability of the SEI layer of Co-NBC@BP@PCA (Fig. 11l). The bond orders of the C–O bond on DME and the C–Cl bond on PCA are the weakest. Therefore, when capturing enriched electrons, these bonds could break first to form DME· and PCA·. It is worth noting that based on the quenching of DME· and PCA· by holes, the excessive decomposition of DME and PCA was obviously terminated with the formation of the organic SEI component because there was a relatively strong van der Waals interaction [147]. Therefore, in a state of rapid charge supply, the hole parts, and the synchronous anion-triggered in situ polymerization of CA enable the elastic SEI to adapt to volume fluctuations, promoted K^+^ transport kinetics, and avoided the excessive decomposition of dimethoxyethane by providing an additional carbon source to generate high specific organic substances. The cracking and reconstruction of working batteries were greatly alleviated, thanks to the in situ binary SEI protective layer with high ionic conductivity and mechanical stability assisted by ultraviolet light, while the non-uniform and vertical K deposition was due to the non-uniformity and mechanical fragility, the lack of a hollow three-dimensional texture, and the extremely expanded planar deposition of the group (Fig. 11m) [148].

An artificially constructed thin SEI layer with uniformly distributed inorganic dihalide components could effectively prevent side reactions between the electrode and the electrolyte, enhance the K^+^ transport ability, and guide the uniform horizontal potassium deposition. In this regard, the fast K^+^ transport electrolyte, the fast K^+^ diffusion ability through the dihalide KF_x_Cl_1-x_, and the enhanced K^+^ transfer ability along the hollow Co-NBC matrix in multiple directions based on the staggered stacking of BP nanosheets greatly promoted the high conversion redox intermediates favorable for KP. It was worth noting that the volume expansion ratios of BP particles and Co-NBC@BP@PCA could be calculated as 1.0% and 0.11%, respectively, which could be obtained from the changes in the in situ XPD profiles along the b-axis of the cell (Fig. 11n). The volume deformation from BP to Co-NBC@BP@PCA was reduced by nearly 10 times, which indeed highlights the high structural stability of Co-NBC@BP@PCA.

To further study the significance of the synergistic effect of electronic structure optimization and SEI layer optimization, the contribution of each component to the overall electrochemical performance of different samples through half-cell experiments was investigated. There are obvious differences among RP, Co-NBC@NBC@BP@CA, and Co-NBC@BP@PCA in terms of the in situ constructed SEI layer and the highly conductive Co-NBC@PCA. It was worth noting that the Coulombic efficiency (CE) of Co-NBC@BP@PCA (99.1%) was much higher than that of Co-NBC@BP@CA (84.3%) and BP (59.6%), thanks to the effective inhibition of harmful side reactions. Meanwhile, on this inorganic-dominated SEI layer, its ionic diffusion ability was relatively high. Co-NBC@BP@PCA also exhibited the most generalized rate and cycling behaviors (Fig. 11o).

### Improving the Electrochemical Stability of the Cathodes

**Fig. 12:**
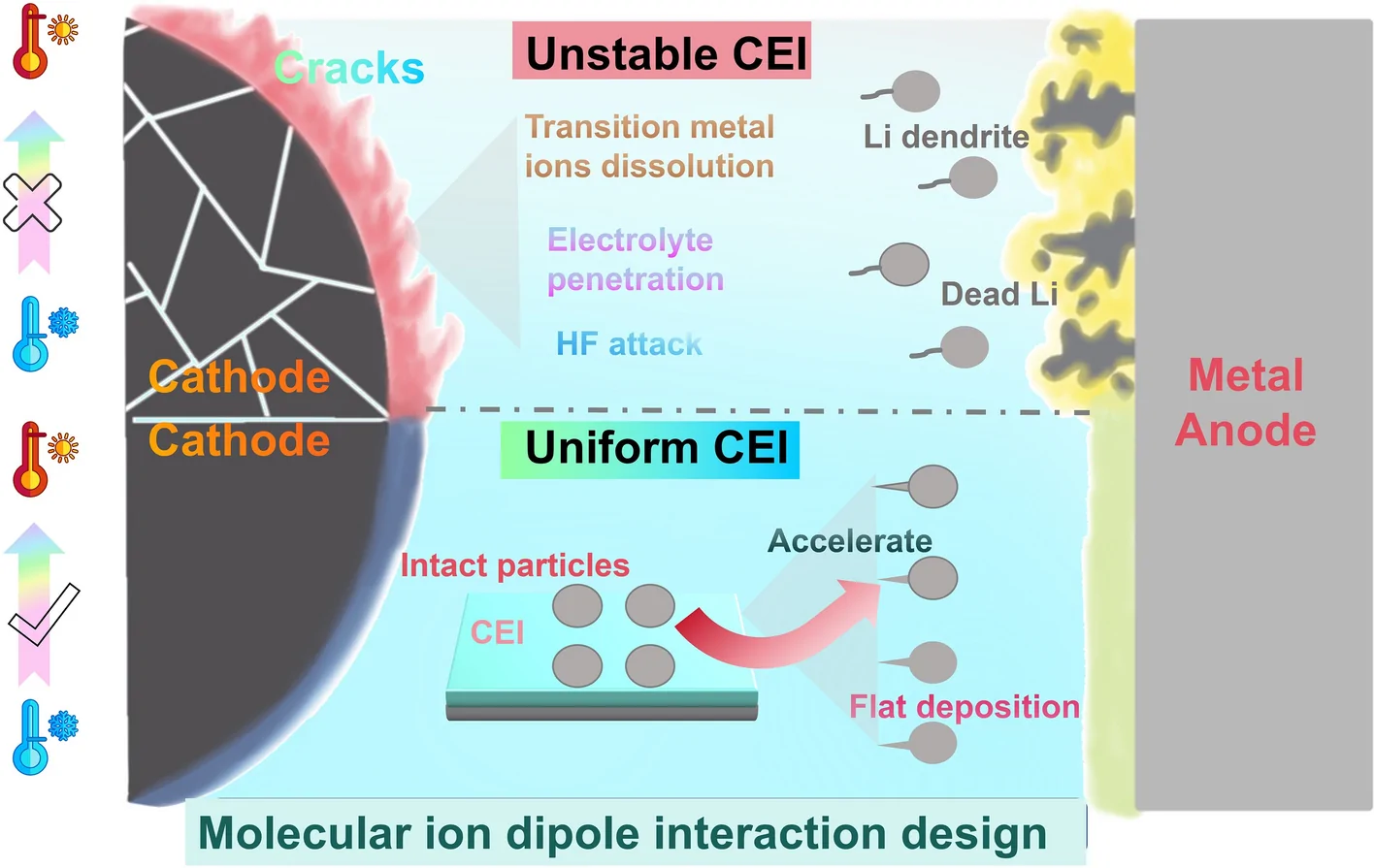
Molecular ion dipole helps improve cathode performance

Moreover, the molecular ion–dipole interaction mechanism could also enhance the electrochemical performance of battery cathodes. For example, it could improve ionic conductivity and build a stable CEI layer [149–151], demonstrating unique and significant influences (Fig. 12). The enhancement of ionic conductivity significantly diminished the resistance during the ionic conduction process, which, in turn, effectively elevated the ionic conductivity of the entire cathode. The CEI layer, serving as a "protective shield" between the cathode and the electrolyte, played a vital role as its quality was closely related to the cycling stability and lifespan of the battery. Hence, it is of great necessity to leverage the molecular ion–dipole interaction mechanism to facilitate the improvement of the electrochemical performance of the cathode.

**Fig. 13:**
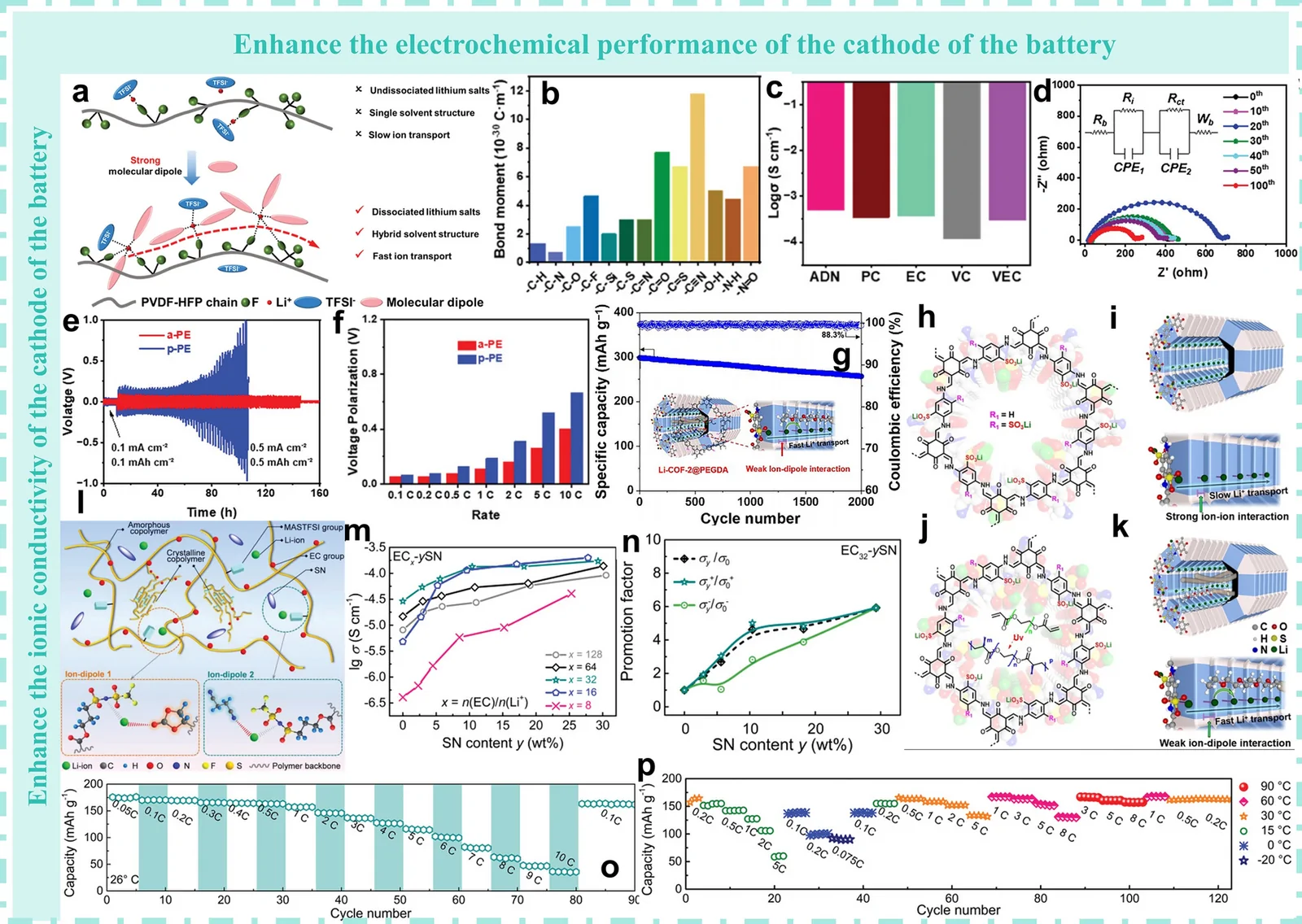
Selection of the molecular dipole. **a** Schematics of solvation structure in PVDF-HFP matrix and molecular dipole for fast Li^+^ transport. **b** Dipole moment of different bonds. **c** Ionic conductivity of the PVDF-HFP-based electrolytes using different molecular dipoles. **d** In situ EIS patterns of the symmetric cell based on the a-PE electrolyte with an equivalent circuit in the inset (Rb: the resistance of polymer electrolyte, Ri: the interface impedance, and Rct: the interface transfer impedanc**e)**. **e** Galvanostatic voltage curves of Li|a-PE|Li and Li|p-PE|Li symmetric cells at 0.5 mA cm^−2^, 0.5 mAh cm^−2^. **f** Voltage polarization bar chart of Li|a-PE|LFP and Li|p-PE|LFP full cells. Reproduced with permission from Ref. [71]. Copyright 2024 Wiley–VCH GmbH. **g** Battery performance diagram. Chemical structure of **h** Li-COF and **i** Li-COF@P_X%_ and conceptual design of their pore functionalization. Li^+^ transport mechanism through the PEGDA-embedded 1D channels in **j** Li-COF and **k** Li-COF@P_X%_. Reproduced with permission from Ref. [154]. Copyright The Author(s) 2024, corrected publication 2024. **l** Schematic illustration of the ion–dipole interactions in the SIPC. Electrochemical properties of SIPCs. **m** Ionic conductivity of the EC_x–γ_SN SIPCs at 25 ℃. **n** Promotion factors of the total ionic conductivity, Li^+^ conductivity, and anionic conductivity in the EC_32–γ_SN SIPCs. Cycling performance of LFP_15_||EC_32–_5.6SN||Li batteries. **o** Rate performance at 26 ℃. **p** Wide temperature operation of the LFP15||EC_32_-5.6SN||Li battery from −20 to 90 C with long cycling at various C-rates. The specific capacity is the discharge capacity. CE represents Coulombic efficiency. Reproduced with permission from Ref. [32]. Copyright 2022 Wiley–VCH GmbH

The excellent degree of ionic conductivity has a tremendous impact on the electrochemical performance of the battery cathode. However, in most metal-ion batteries, there were also different variances in the conductivity of different cathode materials [151, 152]. For example, the conductivity of lithium iron phosphate as a cathode material was relatively less low. Besides, the electrolyte, as an important platform for ionic migration and reaction, also influenced the ionic conductivity. Therefore, it is of great significance to take measures to enhance the ionic conductivity of the cathode and improve the electrochemical performance of the battery. Zhang et al. proposed a strategy to release lithium ions from polymer chains by utilizing ion–dipole interactions. Molecular dipoles with a strong bond dipole moment (–C≡N, 11.8 × 10^−30^ Cm) were introduced into the poly (vinylidene fluoride-co-hexafluoropropylene) (PVDF-HFP) polymer matrix, and an electrolyte with a high ionic conductivity of 5.1 × 10^−4^ S cm^−1^ was obtained at 30° [71]. The results showed that the strong ion–dipole interaction between -C≡N and Li^+^ weakened the ion–dipole interaction between F···Li^+^, promoted the dissociation of Li^+^, and released Li^+^ from the polymer chains. In addition, a hybrid and unsaturated solvation structure was formed with ADN molecular dipoles, PVDF-HFP polymer chains, and TFSI^−^ anions, corresponding to the solvent-separated ion pair (SSIP) solvation structure (Fig. 13a). Consequently, the obtained electrolyte achieved a high ionic conductivity.

Although the solubility of lithium salts is greater in the PVDF-HFP matrix due to its high dielectric constant, Li^+^ interacts with the -F dipoles of the polymer chains and is unable to move, thus affecting the ionic conductivity of the polymer electrolyte [70, 153]. It is necessary to use stronger dipoles for the transportation of Li^+^ in PVDF-HFP-based electrolytes. In the PVDF-HFP chains, the main functional group is –C–F, which showed a lower value (4.64 × 10^−30^ Cm) compared with –C≡N (11.8 × 10^−30^ Cm) and –C–O (7.7 × 10^−30^ Cm) (Fig. 13b). It could be seen that molecules with –C≡N or –C═O dipoles were more likely to interact with Li^+^ through ion–dipole interactions, thereby promoting the dissociation of lithium salts. Moreover, the ionic conductivities of PVDF-HFP-based electrolytes with different molecular dipoles were also measured. As shown in Fig. 13c, the introduction of ADN molecular dipoles significantly improved the ionic conductivity of the electrolyte, reaching 5.1 × 10^−4^ S cm^−1^ at 30 °C, which also confirmed the excellence of ADN molecular dipoles.

After 20 stable cycles, the interface impedance of the assembled symmetric cells decreased significantly in sequence, indicating that the symmetric cells equipped with a-PE materials had a faster ion diffusion rate and better ionic interface conductivity (Fig. 13d). Even when cycled at 0.5 mA cm^−2^ with a capacity of 0.5 mAh cm^−2^, the Li|p-PE|Li symmetric cells could operate for 150 h (Fig. 13e). In contrast, when the current density reached 0.5 mA cm^−2^, the Li|a-pPE|Li symmetric cells suffered a short circuit, which might be due to the poor ionic conductivity. Precisely because of the higher ionic conductivity generated by the dipole interaction, the full cells at different rates exhibited excellent performance. The discharge specific capacities of the Li|a-PE|LFP full cells were 148.6, 145.4, 145.5, 143.5, 0.1, 0.2, 5, 10, 136.7, 128.6, 118.6, and 11.8 mAh g^−1^, which were higher than those of p-PE. When the current density was reset to 0.2, the discharge specific capacity recovered to 138.3 mAh g^−1^ (Fig. 13f). Different from what Zhang reported, Li put forward a new covalent organic framework strategy and proposed a class of solvent-free covalent organic framework single-ion conductors (Li-COF@P) based on weak ion–dipole interactions instead of the traditional strong ion–ion interactions [154], demonstrating excellent electrochemical performance (Fig. 13g). This chemical design has realized a class of solvent-free covalent organic framework single-ion conductors (denoted as Li-COF@P_X%_, where X represents the pore volume utilization, Fig. 13h, i), whose performance was superior to that of previously reported covalent organic framework single-ion conductors. The ion–dipole interactions in Li-COF@P_X%_ are regulated by embedding polyethylene glycol diacrylate (PEGDA) into the pores of the covalent organic framework [155]. The oxygen (O) atoms of the carbon-based groups in the embedded PEGDA allow for ion–dipole interactions with Li^+^ (from the covalent organic framework), transforming the strong dipole interactions into weak dipole interactions and ultimately facilitating ionic dissociation and Li^+^ migration. Therefore, Li-COF@P_X%_ enabled Li^+^ to be easily conducted through the 1 D channels embedded with PEGDA (Fig. 13j, k). In particular, Li-COF-2@P_75%_ exhibited a relatively high Li^+^ conductivity (*σ*_Li+_  = 8.9 × 10^−5^ S cm^−1^) and shows desirable electrochemical performance (Fig. 13g).

Similarly, Li et al. designed a kind of SIPC material [32], which not only improved the conductivity through the design of dipole interaction, but also anchored the transport of anions and promoted the migration of cations through abundant functional groups. This single-ion polymer conductor (SIPC) was well designed through the regulation of ion–dipole interactions, as shown in Fig. 13l. Specifically, the sulfonamide groups in MASTFSILi provided well-dissociated Li^+^, and the anions were covalently bonded to the polymer chains. In the vinylene carbonate (VEC), the –O–(C=O)–O- groups were excellent dipole donors. They could form efficient O–Li^+^ coordination to facilitate Li^+^ conduction, which was similar to the ion–dipole interaction of O…Li^+^ in the polyethylene oxide (PEO)-Li salt systems. Additionally, the –C≡N groups in succinonitrile (SN) were also capable of forming N…Li^+^ coordination with Li^+^ to promote Li^+^ conduction. Through the regulation of these two ion–dipole interactions, the Li^+^ conduction pathways within the single-ion polymer conductor (SIPC) could be optimized.

It could also be seen from Fig. 13m that the σ values of SIPCs increase with the increase in the content of succinonitrile (SN). For example, when the y values were 0, 2.9, 5.6, 10.4, 18.2, and 29.3, respectively, the σ values of the EC_32-y_SN samples were 2.90 × 10^−5^, 5.41 × 10^−5^, 7.79 × 10^−5^, 1.34 × 10^−4^, 1.35 × 10^−4^, and 1.72 × 10^−4^ S cm^−1^, respectively. The increase in SN introduced more ion–dipole interaction sites between SN and Li^+^, resulting in the enhancement of ionic conductivity and thus improving the ionic conductivity of the cathode of the battery. From Fig. 13n, when the SN content increases, the promotion factor for cation conduction in the SIPC system was close to that for the total ionic conductivity, indicating that the addition of SN in the SIPC system mainly enhanced the conduction of cations. Figure 13m also shows that when *y* increases from 2.9 to 10.4, the promotion factor *σ*_γ_^+^/*σ*_0_^+^ increases rapidly. When the σ_Li_^+^ values of EC_32-γ_SN are 2.9, 5.6, or 10.4, their *σ*_Li_^+^ values were greater than 5 × 10^−5^ S cm^−1^. Therefore, by adding a minimum amount of SN, an efficient single-ion conductor-based electrolyte containing –C=O…Li^+^ is obtained. This study provided certain guidance for improving the ionic conductivity of the cathode through dipole interactions and offered a new idea for the design to enhance ionic conductivity.

As shown in Fig. 13o, the LFP15||EC_32–_5.6SN||Li battery had a specific capacity of 170 mA g^−1^ at a low current density below 0.255 mA cm^−2^ (1C, 170 mA g^−1^) and 100 mA g^−1^ at 1.53 mA cm^−2^ (6C). When the load increases, the corresponding LFP35||EC_32–_5.6SN||Li and LFP_50_||EC_32–_5.6SN||Li batteries still exhibited good specific capacities at different C rates. In contrast, due to the low ionic conductivity of the dual-ion polymer conductor (DIPC), the corresponding LFP_15_||5.6DIPC||Li battery could not operate at room temperature (RT). Even at 60 °C, it could only operate at a low current density. During the cycling process, the interfacial resistance was very stable. Even when tested under different harsh temperature conditions and at different high current densities, a high Coulombic efficiency is still maintained (Fig. 13p). The performance of the modified SIPC batteries was still superior to that of batteries made by traditional methods, confirming that the specifically designed SIPC had good lithium-ion conduction and high conductivity through dipole interactions and had great potential in the future battery applications.

### Promoting the Formation of Robust CEI Layer

**Fig. 14:**
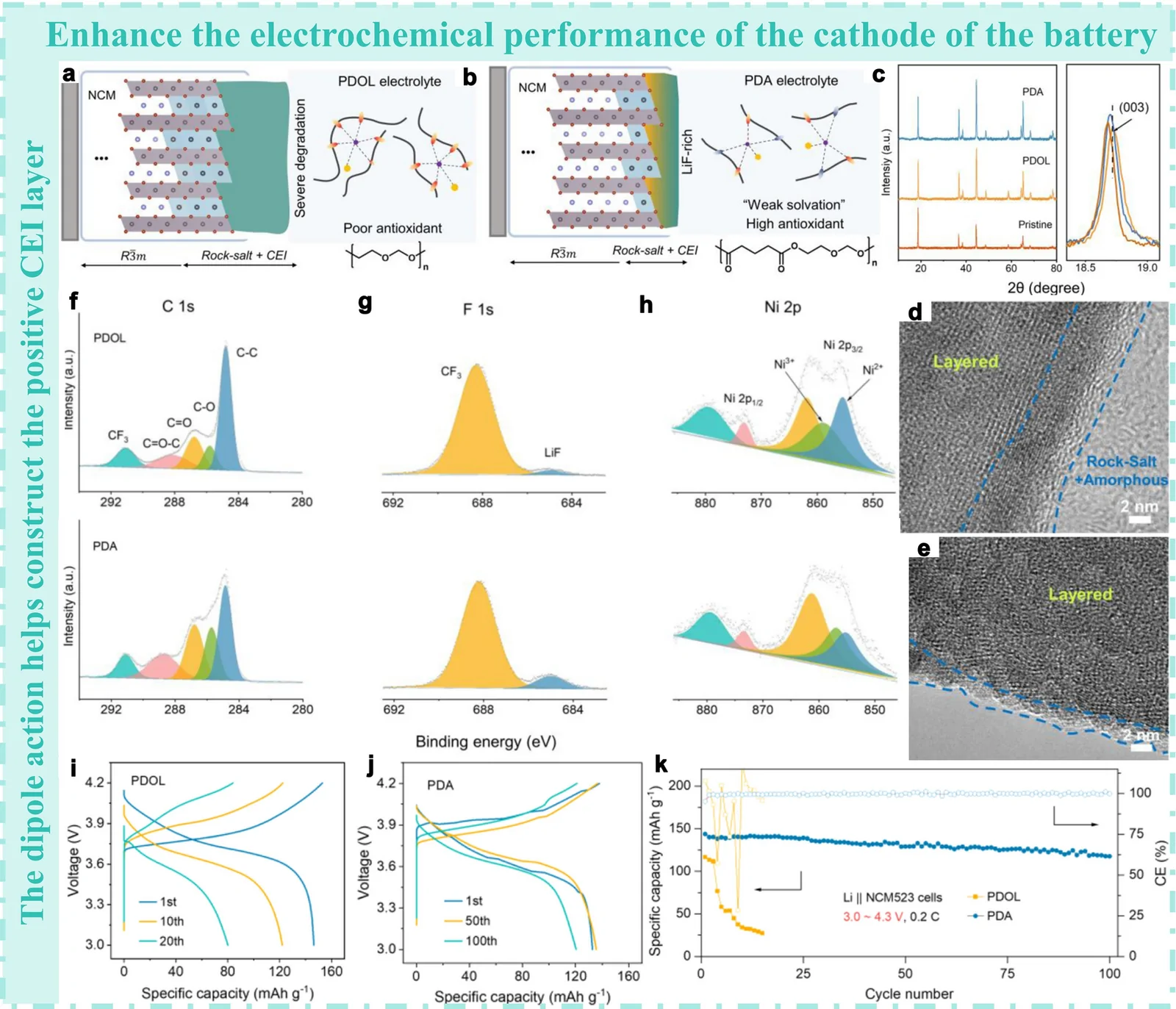
**a** Schematic of interfacial structure of **a** NCM/PDOL electrolyte and **b** NCM/PDA electrolyte, respectively. **c** XRD patterns of NCM523 electrodes. **d**, **e** TEM images of NCM523 particles using studied electrolytes: **d** PDOL and **e** PDA. **f**–**h** XPS spectra of selected elements of the surface on the NCM523 electrodes after cycles in the studied electrolytes: **f** C 1*s*, **g** F 1*s*, and **h** Ni 2*p*. The NCM523 electrodes or particles used for testing were all cycled after 10 cycles in the range of 3.0–4.3 V and at 0.2 C. **i**, **j** Corresponding the voltage profiles of the cells at selected cycles. **k** Cycling performance of Li||NCM523 cells using studied electrolytes at a range of 3.0–4.3 V. Reproduced with permission from Ref. [40]. Copyright 2024 The Authors

Beyond its advantage in enhancing the ionic conductivity of the cathode, the molecular ion–dipole interaction also significantly boosts the electrochemical performance of the cathode by constructing a stable cathode–electrolyte interphase (CEI) layer. Chen et al. designed a system [40]. Under the modulation of ion–dipole interactions, the PDA system had a higher propensity to form a “weak solvation” structure compared to the PDOL system (Fig. 14a, b). This structural feature not only elevated the oxidative stability of the electrolyte to 4.6 V (vs Li^+^/Li), but also yielded a Li^+^ transference number of 0.6 and facilitated the formation of a highly stable LiF-rich CEI layer. Consequently, the Li||NCM523 battery equipped with the PDA electrolyte effectively suppressed polymer degradation under a high-voltage range of 3.0–4.3 V and demonstrated excellent cycling stability. This work furnished essential guidelines for the design of antioxidant polymer electrolytes in high-voltage lithium-ion battery systems.

To further dissect the factors contributing to the enhanced performance of the PDOL electrolyte in the Li||NCM523 battery, the structure and interface of the NCM523 electrode at a cut-off voltage of 4.3 V and a current rate of 0.2C were characterized. Figure 14c illustrates the XRD patterns of the pristine and cycled NCM523 electrodes within the angular range of 10°–80°. The (003) peak of the pristine NCM523 electrode was positioned at 18.68°. After cycling with the PDA and PDOL electrolytes, the (003) peak shifted to 18.70° and 18.73°, respectively. The elevated (003) peak position signified a reduction in the interlayer spacing [156], which implied a higher degree of ion mixing on the NCM523 electrode, a consequence of the severe oxidative degradation of the PDOL electrolyte. Additionally, the surface crystal structure of the cycled NCM523 cathode was characterized using high-resolution transmission electron microscopy (HRTEM). As depicted in Fig. 14d, the surface of the NCM523 particles has undergone a transformation from a layered structure to a mixture of rock-salt and amorphous phases after cycling in the PDOL electrolyte. The thickness of this mixed layer exceeds 7 nm, which acted as a hindrance to the interfacial charge transfer process. Conversely, the degradation of the cathode in the PDA electrolyte was notably mitigated (Fig. 14e), a phenomenon attributed to the protective effect of the CEI layer formed through dipole interactions. Moreover, X-ray photoelectron spectroscopy (XPS) was utilized for analysis (Fig. 14f–h) to characterize the CEI layer of the cycled NCM523 cathode formed in both electrolytes. In the NCM cathode with the PDOL-based electrolyte, the C=O (~ 286.8 eV) and O–C=O (288.3 eV) peaks in the C 1*s* spectrum were predominantly ascribed to the oxidative decomposition products of PDOL. In contrast, for the PDA-based NCM electrode, a portion of the C=O and O–C=O peaks was contributed by the PDA chains bearing ether–ester bifunctional groups. Regarding the F 1* s* spectrum, the presence of lithium fluoride (685.1 eV) indicates the decomposition of LiTFSI on the NCM523 surface at high charging voltages. The higher content of lithium fluoride in the cycled NCM523 cathode with the PDA electrolyte significantly enhanced its interfacial stability. In the Ni 2*p* spectrum, a greater amount of Ni^2+^ was detected on the surface of the cycled NCM cathode containing PDOL, suggesting that the oxidative degradation of PDOL at high voltages was accompanied by the continuous irreversible reduction of high-valence nickel. In contrast, the outstanding antioxidant stability of the PDA electrolyte reduced the Ni^2+^ content on the NCM523 cathode surface and simultaneously improved the structural stability of the cathode interface.

In the electrochemical performance, the Li|PDA|NCM cell outperformed the others by exhibiting a high average CE of 99.7% and remarkable cycling stability with a capacity retention rate of 91% after 100 cycles. Compared with the charge–discharge curves of the Li|PDOL|NCM523 and Li|PDA|NCM523 cells (Fig. 14i, j), the former showed a more distinct increase in polarization. When the cut-off voltage was raised to 4.3 V (Fig. 14k), the Li|PDA|NCM523 cell maintained good cycling stability, while the Li|PDOL|NCM523 cell experienced significant capacity decay and overcharging issues. These superior electrochemical properties could be attributed to the excellent antioxidant stability and rapid Li^+^ transport capability of the PDA electrolyte.

### Preventing Corrosion and Inhibit Side Reactions

**Fig. 15:**
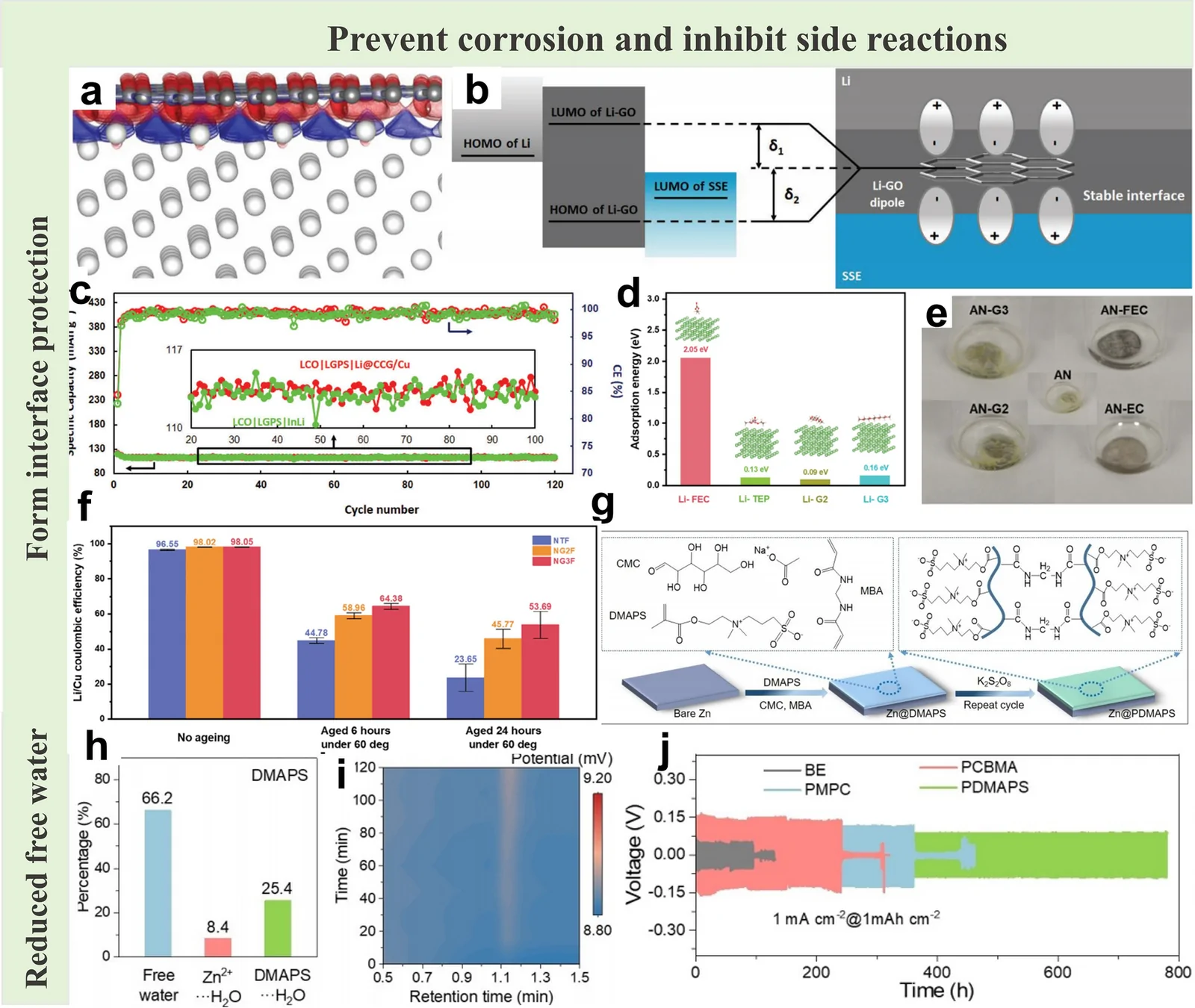
**a** Calculated charge density ρ_diff_ contours between the Li/G interfaces. The isosurface values of red and blue contours are 0.013 (gain charg**e)** and −0.013 e Å^−3^ (loss charg**e)**, respectively. Gray and white spheres represent C and Li atoms, respectively. **b** Schematic representation of the possible increase of HOMO/LUMO energy gap of the Li-GO structure due to the appearance of an interface dipole. **c** Cycling performances of LCO|LGPS|Li@CCG/Cu and LCO|LGPS|InLi. Reproduced with permission from Ref. [159]. Copyright 2020 The Authors. Published by WILEY–VCH Verlag GmbH & Co. KGaA, Weinheim. **d** Calculated adsorption energy between different molecules and lithium metal using density functional theory (DFT). **e** Photographs of Li metal chips soaked in 300 μL different solvents in an Ar-filled glovebox after 24 h. **f** CE of lithium metal in different electrolytes under different aging conditions after 10 cycles. Reproduced with permission from Ref. [68]. Copyright 2024 The Author(s). Advanced Materials published by Wiley–VCH GmbH. **g** Schematic illustration on in situ polymerization of DMAPS on the Zn anodes. **h** Percentages of different forms of H_2_O in the electrolytes. **i** H_2_ evolution in different electrolytes during cycles. **j** Cycling performance of the symmetric cells using different electrolytes. Reproduced with permission from ref [69]. Copyright 2024 Wiley–VCH GmbH

For metal anodes, the design based on molecular ion–dipole interactions is also an effective measure to impede interfacial corrosion and suppress side reactions [157, 158]. Wang et al. designed a dipole interaction structure capable of occupying the energy level orbitals [159], thereby forming a stable interface. This was done with the intention of safeguarding the negative electrode and suppressing the occurrence of side reactions, as depicted in Fig. 15a**.** In this context, the red and blue contours, respectively, signified the gained charge (0.013 eA^−3^) and the lost charge (–0.013 eA^−3^). A charge transfer took place from the lithium surface to the G layer, giving rise to a dipole structure where the Li surface became positively charged and the graphene oxide layer negatively charged. Subsequently, it was demonstrated in this work that this Li-GO dipole structure could remarkably stabilize the Li/solid-state electrolyte (SSE) interface. Moreover, it was found that the emergence of the interface dipole tends to widen the HOMO/LUMO energy gap of Li-GO and extend within the Li and sulfide SSE systems (Fig. 15b). Consequently, the solid-state battery (SSB) utilizing Li@CC/Cu exhibited rather poor cycling stability, suggesting that the side reactions between Li and Li_10_GeP_2_S_12_ (LGPS) might be even more severe compared to those in liquid electrolytes. In sharp contrast, the LiCoO_2_|LGPS|Li@CCG/Cu configuration achieved stable cycling performance. Specifically, at the 120th cycle, it attained a discharge capacity of 112.4 mAh g^−1^, corresponding to a capacity retention rate of 92.9%, with an average coulombic efficiency (CE) exceeding 99.5%. Additionally, the LiCoO_2_|LGPS|InLi system also realized stable cycling, as shown in Fig. 15c. Differing from the approach of Wang et al., Li and co-workers put forward a guiding principle for solvent selection [68], aiming to achieve high Coulombic efficiency, while simultaneously reducing corrosion. Their research was the first to reveal that the dipole moment and orientation of solvent molecules exert a significant influence on the reversibility and corrosion behavior of lithium metal. Solvent molecules possessing a high dipole moment have a greater tendency to adsorb onto the surface of lithium metal, and this adsorption also impacts on the formation and properties of the solid electrolyte interphase. Based on these findings, it was hypothesized that when an electrolyte composed of two or more solvents was in contact with a metal anode, the solvent molecules with the highest dipole moment could account for the largest proportion on the metal surface among all the species present. Subsequently, DFT was employed to compare the interactions between lithium metal and different solvent molecules (Fig. 15d). The results indicated that the fluoroethylene carbonate (FEC) molecule, with its relatively high dipole moment, exhibited the strongest adsorption ability toward lithium metal, boasting an adsorption energy of 2.05 eV. In contrast, other solvent molecules with lower dipole moments displayed significantly weaker adsorption energies. For instance, the adsorption energies of triethyl phosphate (TEP), G2, and G3 were measured as 0.20, 0.09, and 0.16 eV, respectively. The outcomes of molecular dynamics (MD) simulations and DFT calculations jointly suggested that solvent molecules with high dipole moments typically had a strong interaction with lithium metal and were preferentially adsorbed on the metal surface, which was conducive to preventing the occurrence of side reactions.

Therefore, in the modified materials, the different dipole moments associated with various anions do not have a significant impact on the corrosion behavior of lithium metal induced by solvents. This trend could also be observed in AN-based solvents and the other mixed solvents (Fig. 15e). To further verify the influence of the dipole moment on electrochemical corrosion, the above-mentioned LiNO_3_-based electrolyte was utilized to evaluate the retention of lithium metal after aging for different durations. As illustrated in Fig. 15f, at 60 °C, the NG3F electrolyte demonstrated the highest capacity retention rates, reaching 64.38% after aging for 6 h and 53.69% after 24 h. These rates surpassed those of the NTF and NG2F electrolytes, thus validating the efficacy of the proposed strategy.

The presence of free water could modify the solvation structure of the electrolyte. It is combined with solutes like lithium salts to form diverse solvation complexes. Moreover, free water was prone to undergo chemical reactions with certain components within the electrolyte as well as with electrode materials. For instance, in the case of the lithium–metal anodes, water reacts directly with lithium metal to generate lithium hydroxide and hydrogen gas, which leads to the consumption of lithium metal and a reduction in the amount of active material in the negative electrode. Hence, by regulating the content of free water by participating in the reactions within the electrolyte, the occurrence of side reactions can be effectively curbed.

Following this line of thought, Zhang et al. opted to preferentially fix anions and water at different sites of the zwitterionic polymer instead of anions and cations [69]. This approach was adopted to facilitate the free migration of Zn^2+^, thereby reducing side reactions and enhancing the electrochemical performance of the battery. This immobilization process was potentially related to the dipole moment of the zwitterionic polymer. In their work, the zwitterionic polymer was synthesized through in situ polymerization to promote close contact with the zinc foil in the aqueous zinc metal battery (AZMB) (Fig. 15g). Theoretically, these substances could all diffuse toward the vicinity of the zinc anode, potentially triggering the notorious hydrogen evolution reaction (HER). Consequently, there was an urgent need to minimize their free water content as much as possible. As depicted in Fig. 15h, among the three electrolytes studied, the content of free water molecules in PDMAPS was the lowest, accounting for 66.2%. Meanwhile, the content of Zn^2+^-coordinated water remained relatively stable across the three electrolytes, ranging from 8.1% to 8.8%. These results indicated that the combined content of free H_2_O and Zn^2+^-coordinated water in PDMAPS is reduced. Furthermore, PDMAPS exhibited the highest proportion of strong hydrogen bonds, which once again corroborated its strong interaction with water. This finding was consistent with the results obtained from MD simulations. These alterations in the water conformation effectively suppressed HER, a fact that has been directly confirmed by in situ gas chromatography (GC) (Fig. 15i). As a result, the occurrence of side reactions was inhibited. Thanks to these advantages, PDMAPS showed excellent electrochemical performance in the AZMB. Specifically, the symmetric cell employing PDMAPS as the gel electrolyte demonstrated a cycling life of 750 h at a current density of 1 mA cm^−2^ and a specific capacity of 1 mAh cm^−2^ (Fig. 15j), which was significantly superior to that of PMPC (360 h), PCBMA (approximately 240 h), and BE (approximately 90 h). These studies also offered novel ideas and valuable insights regarding the utilization of dipole interactions to protect the negative electrode and mitigate the occurrence of side reactions.

## Conclusion and Outlook

**Fig. 16:**
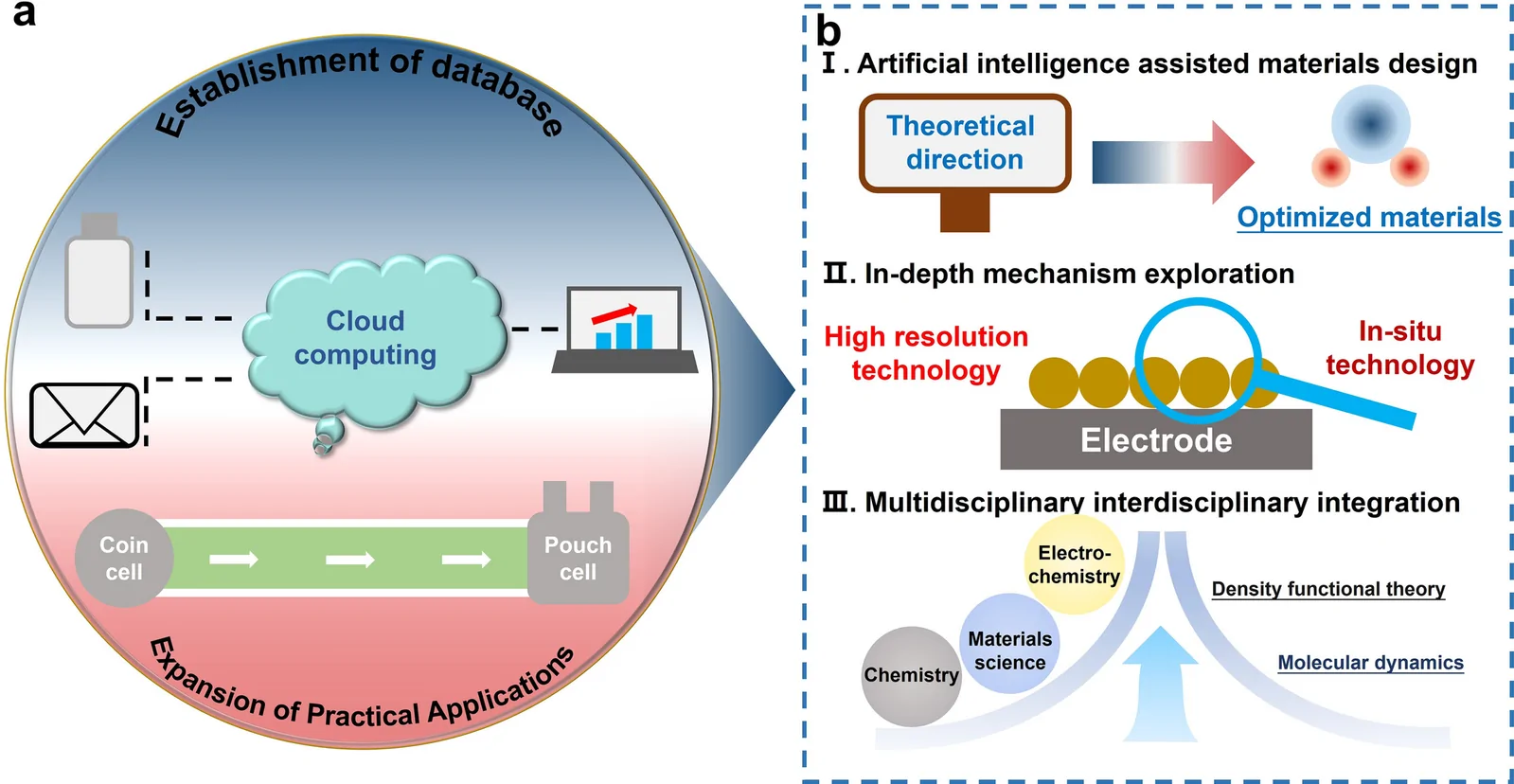
Some perspectives for molecular ion dipoles toward **a** practical applications and **b** research focus in high-energy batteries

Persistent challenges—including metal dendrite formation, low cathode ionic conductivity, electrolyte degradation, anode corrosion, and parasitic side reactions—continue to impede the practical deployment of high-energy batteries. As discussed in this review, molecular and ionic dipole interactions have emerged as an effective strategy to address these limitations, offering a versatile platform to modulate interfacial chemistry, regulate ion transport, and enhance electrochemical stability. This review has comprehensively summarized the mechanisms by which dipole interactions contribute to mitigating these issues, as well as recent advances in their application across diverse battery chemistries. Despite significant progress, the systematic understanding and broader application of dipole interactions in high-energy battery systems remain at an early stage. To accelerate progress, further research efforts are needed to expand the design space, optimize dipole functionalities, and deepen mechanistic insights. In this context, several research directions are proposed to guide future developments (Fig. 16a, b).

### Artificial Intelligence-Assisted Materials Design

With the increasing understanding of molecular and ionic dipole mechanisms, the rational design and synthesis of advanced electrode materials, electrolyte additives, and functional polymer electrolytes incorporating tailored dipolar features will be essential. Integrating dipole characteristics into material structures requires a precise balance of molecular geometry, electronegativity, and dipole moment parameters. To this end, artificial intelligence (AI)-assisted material design tools offer significant promise for accelerating the discovery of materials with optimized dipole-related properties. By leveraging AI-driven screening and predictive modeling, it is possible to fine-tune molecular structures for targeted regulation of dipole interactions at key interfaces. This approach can facilitate the synergistic optimization of electrode, electrolyte, and interfacial components, ultimately enabling the realization of high-energy battery systems with enhanced energy density, prolonged cycle life, and improved safety. Moreover, integrating experimental validation with AI-guided theoretical simulations and building comprehensive databases of dipole-related materials and their performance metrics will further promote the development of next-generation dipole-engineered batteries. These advancements will help meet the escalating demands for high-performance energy storage systems across diverse application sectors, providing critical material and technological support for the broader deployment of high-energy battery technologies.

### In-depth Mechanism Exploration

Although significant progress has been made in applying molecular and ionic dipole interactions to high-energy batteries, a comprehensive understanding of their fundamental mechanisms remains incomplete. Further elucidation of these interactions at the molecular and interfacial levels is essential for guiding the rational design of next-generation battery materials and interfaces. To comprehensively and thoroughly disclose the underlying action mechanisms, it is essential to employ more advanced and precise in situ characterization techniques, like in situ Electrochemical Atomic Force Microscopy and in situ X-ray Photoelectron Spectroscopy. These methods enable real-time monitoring of the dynamic evolution of dipole interactions during battery operation, providing direct insights into how these interactions modulate interfacial microstructures and influence ongoing electrochemical reactions. Through the combination of high-resolution spatial and temporal analyses, it is anticipated that the hidden, dynamic mechanisms of dipole–ion interactions can be systematically unraveled. This deeper mechanistic understanding will offer a robust theoretical framework for the precise control of interfacial chemistry, ultimately enabling the optimization of high-energy battery systems in a more scientifically informed and rational manner.

Besides, in all kinds of batteries with different electrolytes, the dipole interactions may origin from different mechanisms. Although the main mechanism of action is the same, due to the differences in the electrolyte environment and the structure of electrode materials, the effect of the dipole moment varies greatly. Therefore, in the future research, control experiments need to be carried out, such as using different electrolyte systems in the same type of battery or replacing different separators in the same electrolyte system, so as to more comprehensively reveal the mechanism of action of the dipole moment. At the same time, this is also conducive to the targeted selection of optimized materials related to the dipole moment in different batteries.

### Multidisciplinary Interdisciplinary Integration

The exploration of molecular and ionic dipole interactions in high-energy batteries is inherently interdisciplinary, bridging chemistry, materials science, and electrochemistry. The synergy among these fields is essential for deepening mechanistic understanding and accelerating the translation of fundamental insights into practical applications. To advance this field, future research should place greater emphasis on integrating computational modeling with experimental validation. Leveraging the complementary strengths of quantum mechanics-based first-principles calculations, molecular dynamics simulations, and advanced electrochemical characterization techniques will enable high-precision predictions of dipole interaction behaviors and their impacts on interfacial phenomena. Such simulations, when rigorously validated through carefully designed experiments, will ensure the mutual reinforcement of theory and practice, facilitating the rational design of materials and interfaces. Furthermore, incorporating innovative concepts from nanotechnology, bioinspired materials, and soft matter physics into the design of high-energy battery systems can open new pathways for the functionalization and application of dipole interactions. These cross-disciplinary approaches are expected to broaden the application scope of dipole interactions across emerging battery chemistries and provide continuous impetus for technological breakthroughs in high-energy storage systems.

### Establishment of Database

Building a comprehensive and systematic database is crucial to accelerate the mechanistic understanding and practical application of molecular and ionic dipole interactions. Such a database should systematically organize and categorize reported cases, with an emphasis on key parameters including dipole classification criteria, dipole moment ranges, and diverse modes of interaction. By consolidating these data into a well-structured, accessible platform, researchers can efficiently retrieve relevant information, facilitating data-driven material design and accelerating the identification of dipole-related functionalities. Moreover, the establishment of such a resource will highlight the distinctive advantages of dipole interactions compared to other chemical interaction mechanisms, promoting their wider adoption in advanced energy storage systems. Ultimately, this database will serve as a critical reference and decision-support tool for both academia and industry, driving innovation in high-energy battery technologies and supporting the broader development of the energy storage sector.

### Expansion of Practical Applications

Despite significant progress in elucidating the mechanisms and potential applications of molecular and ionic dipole interactions, their deployment in high-energy batteries remains largely limited to the laboratory scale. Bridging this gap and advancing toward large-scale commercialization is now a critical priority. To accelerate this transition, closer collaboration between academia and battery manufacturers is essential. By leveraging industrial expertise in large-scale production, joint efforts should focus on optimizing manufacturing processes, addressing challenges related to cost control, scalability, and process stability. These measures will ensure that dipole-engineered battery systems not only meet high-performance standards, but also demonstrate commercial viability and competitiveness in the market. Furthermore, coordinated efforts should be made to promote the integration of such optimized battery technologies into real-world applications, including electric vehicles and grid-scale energy storage systems. Such efforts will enhance energy storage efficiency and contribute to the realization of sustainable energy goals. Ultimately, facilitating the translation of scientific breakthroughs into practical technologies will generate broad societal and economic benefits, supporting the global transition to cleaner and more efficient energy systems.
